# The impact of air transport availability on research collaboration: A case study of four universities

**DOI:** 10.1371/journal.pone.0238360

**Published:** 2020-09-04

**Authors:** Adam Ploszaj, Xiaoran Yan, Katy Börner

**Affiliations:** 1 Centre for European Regional and Local Studies EUROREG, University of Warsaw, Warsaw, Poland; 2 Indiana Network Science Institute, Indiana University, Bloomington, Indiana, United States of America; 3 School of Informatics, Computing, and Engineering, Indiana University, Bloomington, Indiana, United States of America; IT University of Copenhagen, DENMARK

## Abstract

This paper analyzes the impact of air transport connectivity and accessibility on scientific collaboration. Numerous studies demonstrated that the likelihood of collaboration declines with increase in distance between potential collaborators. These works commonly use simple measures of physical distance rather than actual flight capacity and frequency. Our study addresses this limitation by focusing on the relationship between flight availability and the number of scientific co-publications. Furthermore, we distinguish two components of flight availability: (1) direct and indirect air connections between airports; and (2) distance to the nearest airport from cities and towns where authors of scientific articles have their professional affiliations. Based on Zero-inflated Negative Binomial Regression, we provide evidence that greater flight availability is associated with more frequent scientific collaboration. More flight connections (connectivity) and proximity of airport (accessibility) increase the expected number of coauthored scientific papers. Moreover, direct flights and flights with one transfer are more valuable for intensifying scientific cooperation than travels involving more connecting flights. Further, analysis of four organizational sub-datasets—Arizona State University, Indiana University Bloomington, Indiana University-Purdue University Indianapolis, and University of Michigan—shows that the relationship between airline transport availability and scientific collaboration is not uniform, but is associated with the research profile of an institution and the characteristics of the airport that serves this institution.

## Introduction and prior work

Despite the proclaimed “death of distance” [1, 2], geography is of constant importance for scientific collaboration [3–5]. Numerous studies demonstrated that the likelihood of collaboration declines with growing distance between prospective collaborators. This effect is observed both at the micro level of buildings or campuses, as well as at the macro level of collaboration networks among cities, regions, and countries.

At the micro level, Allen [6] showed in the 1970s that the frequency of communication between individuals in science and engineering organizations drops exponentially with the growing distance between their offices. Subsequent research revealed that collaboration is more likely not only between closely sited or collocated individuals [7, 8] but also between those whose daily paths cross frequently or largely overlap [9, 10].

At the macro level—where distance is measured in kilometers rather than meters—a large body of evidence indicates the negative impact of spatial separation on research collaboration: the greater the distance, the lower the likelihood of collaboration. Furthermore, geographical distance not only decreases the likelihood of any collaboration, but also reduces the intensity of collaboration, as measured by the number of co-publications, co-patents, and collaborative projects [11–13]. The relationship between distance and collaboration is frequently analyzed in the framework of the general gravity model [14–26]. The gravity model is conceptually based on Isaac Newton’s law of gravitation [27–29]. It says that the gravitational force between two objects is proportional to their masses and inversely proportional to the square of the distance between them. The model assumes that not only the distance between collaborating units matters, but also their “masses” should be taken into account. Here “mass” refers to research capacity of the collaborating units, typically measured by research and development employment or expenditures, as well as by accumulated research outputs: stocks of funded projects, publications, and patents. The gravity model applied to scientific collaboration clearly shows that the probability and intensity of research collaboration are negatively related to the geographic distance which separates the units in question and are positively affected by their accumulated research potential [20–26].

The detrimental effect of geographical distance on the likelihood of research collaboration remains significant even when controlling for important features of collaborating units, type of collaborative relations, and the context in which collaboration occurs. Previous studies controlled for scientific quality, most frequently measured via citations [30–32], differences in cooperation patterns accross various fields of science [33–36], type of research [37], and the type of collaboration data used in the analysis, such as co-publications, co-patents, and collaborative projects [38, 39]. Prior work has also considered different types of non-spatial proximities, including cognitive, cultural, economic, institutional, organizational, social, and technological [40–44].

The rise in research collaboration manifests itself not only in the growing number of co-authors per paper (and co-inventors per patent), but also in the increasing co-authorship among authors whose institutional affiliations were in different countries. Between 1990 and 2011, the percentage of internationally co-authored papers indexed in the Science Citation Index increased from 10.1% to 24.6% [45]. Co-authorship is particularly intense between authors affiliated with the largest research centers, which serve as major hubs in the global scientific cooperation network [46, 47]. At the same time, researchers are increasingly collaborating across greater distances. Between 1980 and 2009 the mean collaboration distance per publication raised from 334 to 1,553 kilometers [48].

The distance between collaborating units in spatial scientometrics studies is usually measured as geographical distance along the surface of the earth (“as the crow flies”), between points which are defined by geographical coordinates: latitude and longitude [49]. The actual accessibility is taken into account surprisingly rarely in empirical studies of scientific collaboration. To our best knowledge, only following empirical works considered actual transport accessibility as a covariate of scientific collaboration. Andersson and Ejermo [50] included road travel time in their case study of Swedish patent co-authorship network. Ejermo and Karlsson [51] studied road and air travel time impact on co-patenting in Sweden. Frenken and colleagues [52] analyzed the relationship between the number of co-publications and road travel time at regional level in the Netherlands. Ma, Fang, Pang, and Li [53] hypothesized that high-speed railway accessibility can be one of the factors explaining the intensity of scientific cooperation between Chinese cities. Later, the hypothesis was supported with evidence from instrumental variable regression study designed by Dong, Zheng, and Kahn [54]. Furthermore, Hoekman, Frenken, and Tijssen [21] argued that European regions with a major international airport are more likely to develop intensive international scientific collaboration. Against this background, the study of Catalini, Fons-Rosen, and Gaulé [55] stands out as the authors used a quasi-experimental design (natural experiment) to examine the impact of introducing a new, low fare, air route on the probability of scientific cooperation. Their analysis focuses on 890 faculty members in chemistry departments of research-intensive US universities in the period from 1991 to 2012. The results show that the introduction of new routes significantly increases the likelihood of collaboration among US chemistry scholars. The greatest impact is observed in the case of early career scholars, who usually have fewer resources than established professors do, and therefore cheaper flights may be more important to them.

Our study extends prior work by analyzing the relationship between scientific collaboration and worldwide air transport availability. We distinguish two components of flight availability: (1) direct and indirect air connections between airports (connectivity), and (2) distance to the nearest airport (accessibility) from cities and towns where scientific articles are affiliated. We test the hypothesis that better air transport connectivity and accessibility—ceteris paribus—is positively associated with scientific collaboration. Furthermore, we hypothesize that the relation depends on research capacity and profile of a given university and the flight network of an airport that serves the university. To check if such heterogeneity exists, we based our analysis on a non-random sample of four purposively selected universities and their matching airports; or more precisely: four co-authorship networks of four universities and flight networks of airports that are the default airports for researchers working at these universities.

It should be underlined that the purpose of this analysis is not to examine the full set of factors affecting collaboration in science, such as specialization and the division of scientific labor, growing interdisciplinarity, exorbitant costs and complexity of big science, personal characteristics and preferences, academic mobility, collaboration-focused science policy, or long-term inter-organizational relationships, among others. These topics have been comprehensively covered in numerous publications; see, for example, a classical study by Katz and Martin [56] and recent comprehensive reviews of the topic [5, 57–59]. Instead, we aim to answer one question: does better air transport connectivity between potential collaborators constitute a statistically significant factor that increases the probability of collaboration as measured by the number of co-authored papers. We apply regression based cross-sectional analysis to examine how the differences in air transport availability and accessibility correlate with the number of co-authored papers while controlling for the known critical factors influencing collaborative behavior at the aggregated spatial level, i.e., geographic distance and accumulated scientific capacity (as in prior works mentioned above).

The remainder of the paper is organized as follows. The next section introduces our empirical strategy, sample section, variables and descriptive statistics. Subsequently, we present our approach to model the relation between the number of co-authored papers and air transport availability. We then discuss findings. The paper concludes with discussion and conclusions. Supporting information includes detailed information on data sources and data processing procedures, as well as information needed to replicate the results of this study.

### Empirical strategy and descriptive statistics

In this analysis we employ the ego network approach, i.e., we analyze spatial relations between a focal node—“ego”—(in our case: a university’s geolocation) and the nodes to whom the ego is related—“alters”—(in this case other cities and towns listed as affiliations by co-authors). Although our analysis would be possible based on a single ego network, we opted for having four ego networks. This tactic allows, on the one hand, to increase the statistical power of the analysis, and on the other, to identify possible heterogeneities in particular cases. We purposefully selected four universities that share some characteristics and vary in others. To ensure comparability of the analyzed cases, we assumed that the egos would be selected from the pool of comprehensive research-intensive public universities in the U.S. The central selection criterion was the possibility of the unambiguous assignment of a university to a single airport that can be considered as a “default” option for air travel for scholars affiliated with the university. For this reason, we disqualified universities localized in metropolitan areas served by two or more major airports with commercial flights (such as New York, Chicago, Bay Area, etc.). In the next step, we considered airports with different levels of air network development and in consequence various levels of passenger traffic. On this basis, we selected four university-airport pairs. The final analytic sample comprised of Arizona State University at Tempe (ASU), Indiana University Bloomington (IUB), Indiana University-Purdue University Indianapolis (IUPUI) and the University of Michigan at Ann Arbor (UMICH) (only the main campuses were included in the study). UMICH is served by Detroit Metropolitan Airport (DTW) and ASU by Phoenix Sky Harbor International Airport (PHX). Both DTW and PHX are important hubs. According to Federal Aviation Administration data, PHX was the 11th US airport in terms of the number of passengers in 2016 (including 3.1% of passengers that used PHX’s reliever airport Phoenix–Mesa Gateway Airport), while DTW took 18th position. IUB and IUPUI constitute a special case. The two campuses are served by the same airport, Indianapolis International Airport (IND). IND is an airport with considerably less passenger traffic than PHX and DTW. In 2016, it was 46th U.S. airport in terms of the number of passengers. As a result of the selection procedure, our research sample is composed of arguably comparable universities unambiguously assigned to default commercial airports which somewhat differ in the roles they play in the U.S. air transport network. Our selection procedure, does introduce some undesirable properties; as with any non-random sampling research, the results presented in what follows cannot be interpreted as describing any population beyond our sample. For this reason, we treat this exercise as a case study. However, in future studies the methods used in this paper could be applied to a larger set of institutions, possibly the entire set of all research active institutions—given data availability.

Each of the four constructed ego-networks is multidimensional, which means that an ego and its alters are related by more than one type of relation. In this study, the key relations are the co-authorship of scientific papers, air transport connections, as well as the geographic distance between an ego and its alters. In addition to variables that characterize links, our dataset also includes variables that characterize nodes (both egos and alters): number of research papers published in a given node (“scientific mass”), and the distance from a given node to its default airport.

The number of co-authored papers is the dependent variable in this study. Co-authorship were identified on the basis of the co-occurrence of author affiliations in articles published in years 2008–2013 and indexed in the Web of Science database. We employed the full counting method, i.e. each co-authored paper is counted as one for a given ego-alter relation, regardless of the number of authors, organizations, geo-locations or countries involved [60]. The advantage of this approach—as compared to fractional counting—is the intuitive interpretation of results, as well as the possibility of using well-established statistical models for event counts data [61].

**Fig 1:**
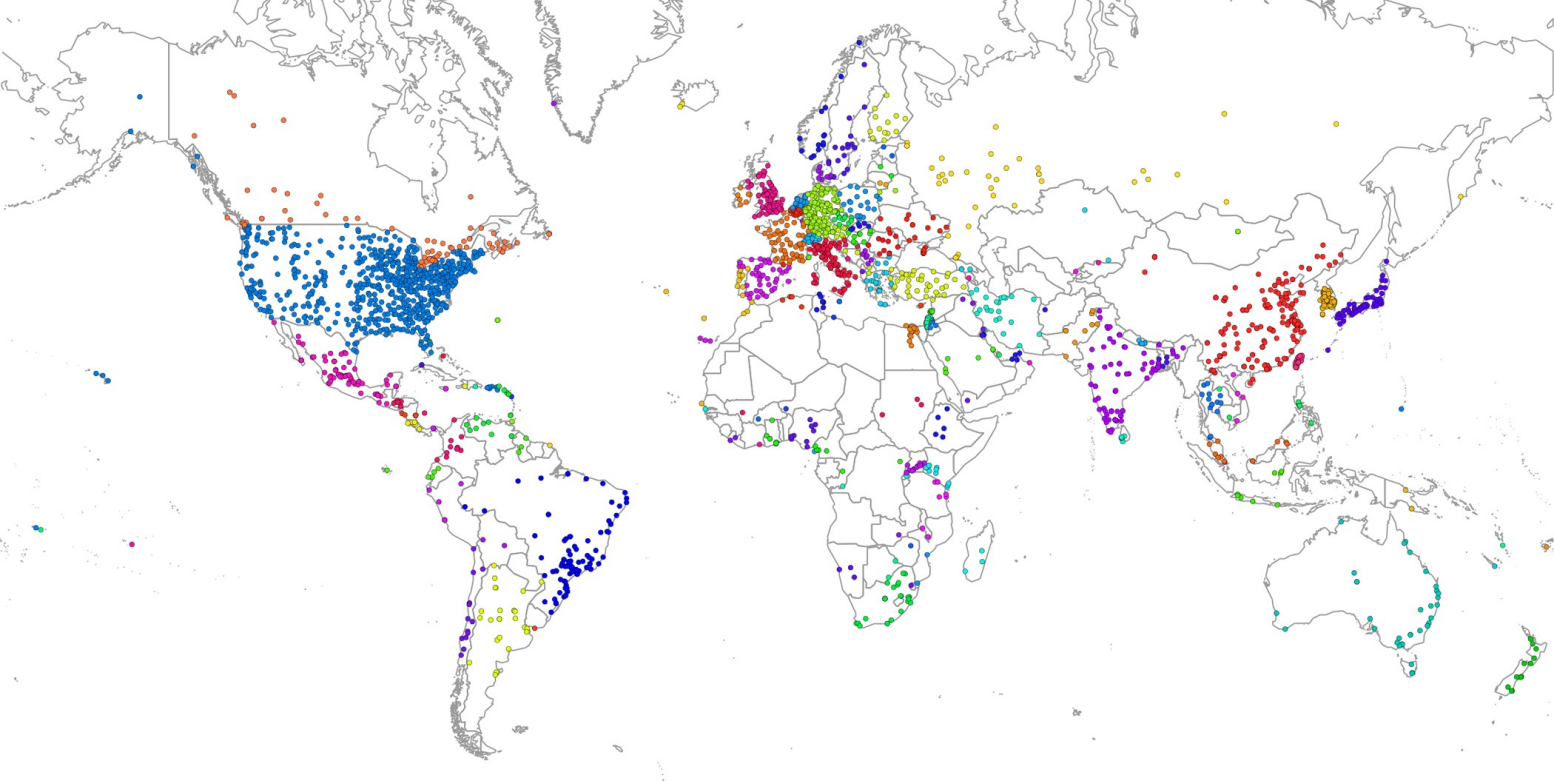
Merged cities and metro areas under this study. *Colours represent countries.

The dependent variable is measured for each of four institutions—ASU, IUB, IUPUI, and UMICH—as the number of co-authored papers between the given campus and various geographical units across the globe (henceforth called as ‘destinations’). To ensure coherence and international comparability geo-locations are merged into 2,245 town/city/metropolitan/regional entities, such as European NUTS2 regions and US Metropolitan Statistical Areas (see Fig 1). For each of four selected universities a separate egocentric co-authorship network was constructed. In consequence, we obtained four ego-networks, in which an ego was ASU, IUB, IUPUI or UMICH, and alters (destinations) were spatial units from around the world (for the details on data sources and data processing, please refer to the S1 File).

To measure air transport availability we employed a number of variables grouped into two categories: commercial air transport connectivity and transport accessibility to the nearest airport. The accessibility variable is measured as the geographical distance from the center (centroid) of a destination to its nearest airport with commercial flights. To account for connectivity, we tested three approaches. The most straightforward variable is a ‘Minimum number of stops to reach destination’. This factor variable is based on a minimum number of connecting flights needed to travel from ego’s nearest airport to the airport nearest to the centroid of destination geographical unit. It is measured up to 4 connecting flights (or 3 stops) and takes values: 0 (for direct flights), 1, 2, or 3. Second measure ‘LinesXstop’ takes into account number of flights between ego and destination airports. ‘Lines0stop’ accounts for direct flights only. ‘Lines1stop’ measures direct and indirect flights up to one stop (i.e., up to two connecting flights). ‘Lines2stop’ considers direct and indirect flights up to two stops, while ‘Lines3stop’ adds connections requiring 3 stops. To take into account the preference for flights with fewer transfers, weights are applied: 1 for direct flights, 0.5 for one stop connections, 0.33 for two stop, and 0.25 for three stops. ‘SeatsXstop’ variable is constructed in a similar way, but it also takes into account number of seats available on direct and connecting flights. The use of concurrent connectivity variables aims to better understand the relationship between air transport and scientific collaboration. Three questions are particularly interesting in this case. First, are direct connections more important than connecting flights? Second, are indirect flights with fewer stops more important than those with more stops? Third, does the passenger capacity (number of available seats) matter?

Three control variables are used in this study. ‘Geographical distance’ between an ego-institution and a destination is measured along the surface of the earth. The literature suggests that geographical distance alone explains some variation in scientific collaborations. However, we hypothesize that models accounting simultaneously for geographical distance and flights availability variables will fit the data better. The second control variable is the ‘Number of papers at destination’. This variable can be seen as the equivalent of a mass term in the gravity model approach. We assume that probability and intensity of collaboration between ego and destination depend primarily on the scientific capacity of a destination. Collaboration with city, region, or country that have virtually no research activities is improbable. While collaboration with global knowledge hubs, e.g. Oxford, Paris, or Tokyo, can be intensive, despite the geographical distance. The third control variable is a ‘Disciplinary similarity’ measured as the cosine coefficient expressed in percentages [62] and based on papers’ classification into 13 broad disciplines as defined in [63] and [32]. It measures the degree of disciplinary similarity between the collaborating places. ‘Disciplinary similarity’ ranges between 0 (completely different disciplinary structure) and 1 (identical disciplinary structure). Greater disciplinary similarity usually goes in hand with more collaboration [12, 64].

Our full dataset of 8,980 observations (units of analysis) consists of four institutional sub-datasets, each comprising 2,245 observations (see Tables 1 and 2). An observation is defined as a multidimensional link (co-authorships, geographical distance, air links, etc.) between university campus in question—one of the four ego-institutions—and one of 2,245 geographical entities around the world that have at least one paper affiliated as identified by Mazloumian et al. [32]. The number of co-authored papers between ego-institution and defined geographical entities—the dependent variable in this study—ranges from 0 to 3433, with the mean value of the variable equal to 15.4 (in the period of 2008–2013). It means that the four analyzed institutions co-authored on average 15.4 papers per possible relationship between the institution and one of the defined geographical units. In this regard, UMICH stands out from the other three universities. Its average number of papers co-authored with researchers affiliated with institutions located in other spatial units around the world equals 34.6, while for other institutions it lays in the range from 8.2 to 9.9.

**Table 1:** Descriptive statistics–Full dataset.

Variable	Observations	Mean	Std. Dev.	Min	Max
Number of co-authored papers	8980	15.4	89.5	0	3433
Geographical distance (mi)	8980	4232.3	2669.4	20.4	11171
Number of papers at destination	8980	5373.3	13866	1	201693
Disciplinary similarity	8980	63.8	25.9	0.6	99.8
Distance to airport at destination (mi)	8980	24.8	25.4	0.4	327
lines0stop	8980	0.1	0.7	0	15
lines1stop	8980	3.8	6	0	55
lines2stop	8980	18	16.8	0	127
lines3stop	8980	114.6	91.6	0	822
seats0stop	8980	24.1	128.5	0	2016
seats1stop	8980	623	1049.3	0	8523
seats2stop	8980	3071.3	3002.3	0	21249
seats3stop	8980	95361.2	153682.8	0	1535855
Min. number of stops to destination	8980	1.5	0.7	0	4

**Table 2:** Descriptive statistics–Institutional sub-datasets.

Variable	Observations	Mean	Std. Dev.	Min	Max
**ASU**					
Number of co-authored papers	2245	9.9	40.8	0	793
Geographical distance (mi)	2245	4762.6	2619.2	82.9	10934
Number of papers at destination	2245	5375.3	13875.2	1	201693
Disciplinary similarity	2245	70.3	20.6	13.3	98.3
Distance to airport at destination (mi)	2245	24.8	25.4	0.4	327
Lines0stop	2245	0.3	1	0	15
Lines1stop	2245	4.4	7.3	0	55
Lines2stop	2245	20.6	19.4	0	127
Lines3stop	2245	131.7	104.7	0	822
Seats0stop	2245	42.4	185.7	0	2016
Seats1stop	2245	759.1	1300.9	0	8523
Seats2stop	2245	3653.5	3495.3	0	21249
Seats3stop	2245	127024.4	182298.8	0	1521228
Min. number of stops to destination	2245	1.4	0.7	0	4
IUB*					
Number of co-authored papers	2245	8.2	30.6	0	469
Geographical distance (mi)	2245	4085.3	2684.7	20.4	11075
Number of papers at destination	2245	5380.2	13879.6	1	201693
Disciplinary similarity	2245	68.1	18.6	8.3	97.3
Distance to airport at destination (mi)	2245	24.8	25.4	0.4	327
Lines0stop	2245	0	0.4	0	9
Lines1stop	2245	2.8	4.8	0	37
Lines2stop	2245	15	14.1	0	95
Lines3stop	2245	95.7	76.6	0	648
Seats0stop	2245	6.3	49.7	0	1115
Seats1stop	2245	419.4	737.2	0	4937
Seats2stop	2245	2375.4	2253.6	0	13831
Seats3stop	2245	60251.8	120158.7	0	1105416
Min. number of stops to destination	2245	1.6	0.7	0	4
IUPUI*					
Number of co-authored papers	2245	9.1	44.6	0	822
Geographical distance (mi)	2245	4080.4	2683.5	40.5	11095
Number of papers at destination	2245	5375.8	13875.8	1	201693
Disciplinary similarity	2245	51.3	31.3	0.6	99.8
Distance to airport at destination (mi)	2245	24.8	25.4	0.4	327
Lines0stop	2245	0	0.4	0	9
Lines1stop	2245	2.8	4.8	0	37
Lines2stop	2245	15	14.1	0	95
Lines3stop	2245	95.7	76.6	0	648
Seats0stop	2245	6.3	49.7	0	1115
Seats1stop	2245	419.4	737.2	0	4937
Seats2stop	2245	2375.4	2253.6	0	13831
Seats3stop	2245	60251.8	120158.7	0	1105416
Min. number of stops to destination	2245	1.6	0.7	0	4
**UMICH**					
Number of co-authored papers	2245	34.6	164.2	0	3433
Geographical distance (mi)	2245	4000.7	2619.9	30.4	11171
Number of papers at destination	2245	5361.7	13842.6	1	201693
Disciplinary similarity	2245	65.5	26.7	5.7	99.5
Distance to airport at destination (mi)	2245	24.8	25.4	0.4	327
Lines0stop	2245	0.2	0.8	0	9
Lines1stop	2245	5	6.5	0	50
Lines2stop	2245	21.4	18	0	122
Lines3stop	2245	135.2	97.1	0	805
Seats0stop	2245	41.3	159.4	0	1295
Seats1stop	2245	894.2	1204.7	0	8396
Seats2stop	2245	3880.8	3424.7	0	21114
Seats3stop	2245	133916.5	165648.9	0	1535855
Min. number of stops to destination	2245	1.2	0.7	0	4

* IUB and IUPUI are served by one airport, Indianapolis International Airport (IND), therefore they have the same values of air transport variables.

**Fig 2:**
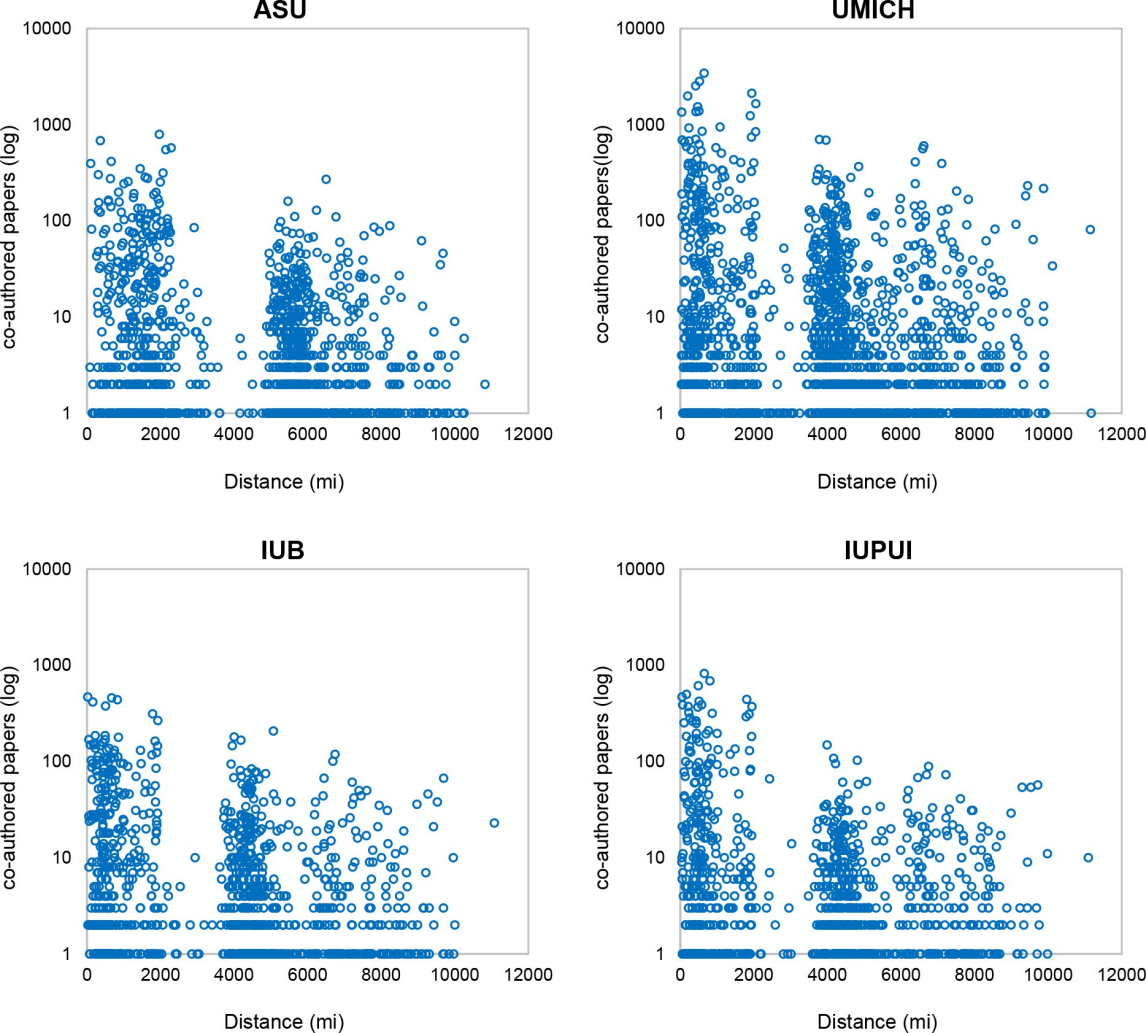
Co-authored papers distribution by geographic distance.

The geographical distance between the four ego-institutions and their collaborators varies from 20.4 to 11,171 miles. Mean geographical distance between all possible dyads (between one of the four ego-institutions and all other possible collaborators in their network) is 4,232 miles. To put this number in context, recall that the distance between New York City and Los Angeles is about 2,450 miles. The high average geographical distance results for the fact that many coauthors have institutional homes on other continents. UMICH has the lowest mean geographical distance between it and collaborating institutions (4,001 miles), followed by UIPUI and IUB (4,080 and 4,085 miles respectively), while ASU is characterized by the highest geographical separation from its collaborators (4,763 miles). The juxtaposition of the number of co-authored papers and the distance between co-authors’ affiliations reveals that collaboration is not uniformly distributed across geographic space (see Fig 2). A pattern is evident across all four institutions: A university substantial proportion of collaborations take place in the range up to 2,000 miles, there are almost no collaborations in the 2,000 to 4,000 mile range, then, from over 4,000 miles (over 5,000 miles in the case of ASU) collaborations are again evident. Comparing these distances to a map shows that the closest set of collaborations reflects those in which the collaborator is within the continental U.S. or North America, the gap at 2,000 to 4,000 miles reflects the Atlantic and Pacific Oceans, and the range from 4,000 to 6,000 miles reflects mainly U.S.—European collaborations.

Descriptive statistics of ‘Number of papers at destination’ and ‘Distance to airport at destination’ are almost identical for the full dataset and each of institutional datasets. This is due to the fact that each university has the same set of possible collaborators, except for itself—i.e., ASU ego network excludes ASU, IUB ego network excludes IUB, etc. The number of papers at destination was as low as one (recall that only geographical entitles with at least one affiliated paper were included in the dataset), and as high as almost 202 thousand (Boston metropolitan area). The mean distance from collaborating destination to its nearest airport was about 25 miles. The longest distances to the nearest airport with scheduled flights occur in vast and sparsely populated countries, such as Russia or Canada, and in emerging economies, mainly in Africa and South America.

The values of air transport connectivity variables vary substantially among the four institutional sub-datasets. Three airports that serve four considered campuses—note that IUB and IUPUI are served by a single airport, IND, located on the outskirts of Indianapolis—differ regarding the number of direct flights to collaborative destinations. Consequently, they also differ in the number of collaborating destinations that reachable by direct flights, as well as flights with one, two or three stopovers/connections. UMICH is served by Detroit Metropolitan Airport (DTW) and has a privileged position owing to the fact that scholars from Ann Arbor can reach collaborators in 301 different collaborating destinations via direct flights. Phoenix Sky Harbor International Airport (PHX) serves ASU and provides direct connections to 218 destinations, whereas IND airport only provide 53 direct-flight-accessible destinations. Furthermore, UMICH scholars can travel to more destinations using one-stop connecting flights than scholars from other three universities. On the other hand, for ASU, IUB and IUPUI researchers more destinations are available only via connecting flights with at least two stops (see Table 3). As a result, air transport connectivity variables—‘LinesXstop’, ‘SeatsXstop’, and ‘Minimum number of stops to destination’—have higher values for UMICH, than in the case of ASU and, in particular, IUB and IUPUI (see Table 2).

**Table 3:** Destinations reachable with direct and connecting flights from airports serving four studied universities.

Airport	Direct	1 stop	2 stops	3 stops	Total
Detroit (DTW)	301	1255	658	31	2245
Indianapolis (IND)	53	894	1134	164	2245
Phoenix (PHX)	218	913	1042	72	2245

DTW serves UMICH, IND serves IUPUI and IUB, while ASU is served by PHX (the data for PHX includes ist reliever airport Phoenix–Mesa Gateway Airport).

Variables used in this study are not distributed normally (see figures presented in S1 File). Most of variables is right-skewed. Our outcome variable, the number of co-authored papers, is the extreme example: it is highly right-skewed with excessive number of zero-valued observations. Therefore, it is crucial to choose the right method to properly model this data.

### Modeling approach

To model the impact of air transport availability on scientific collaboration we employed a zero-inflated model—i.e., one that appropriately accommodates non-normally distributed data with frequent zero-valued observations. This class of models initially developed by Mullahy [65] and extended by Lambert [66] and Greene [67] is designed for event count data where the dependent variable follows a zero-inflated probability distribution [68, 69] and has been applied in numerous scientometrics studies [70–74]. Our analytic dataset fits the requirements for using these models perfectly—about 45% of the outcome variable equals zero. That is, during the observed period, the four ego-institutions had no co-authorships with 45% localizations that are identified as having published at least one scientific paper (according to data from 32). The zero-inflated model assumes that zero outcome can result from two different processes. First, the absence of collaboration can be due to the lack of research capacities at the destination. In this case, the expected outcome is zero. Second, if the destination has some research capacities, it is then a count process. Zero outcome is still possible (e.g. due to different research profiles), but numerous co-authorships are very likely.

Consequently, the zero-inflated model has two components: “inflate” part that accounts for excess zeros (the equivalent of logit model) and a proper “count” part. In the count part, we used three control variables—i.e. ‘Geographical distance’, ‘Number of papers at destination’, and ‘Disciplinary similarity’—and independent variables for air transport connectivity and accessibility. To construct inflate part we used a single predictor: ‘Number of papers at destination’. This decision is based on the assumption that the adequate critical mass of scientific capacity determines the emergence of scientific collaboration, regardless of geographical distance and transport accessibility. To ensure robustness of the analysis, we tested several other specifications that included other variables in the 'count' part of the model. These additional variables do not significantly change the values ​​of the coefficients or the overall fit of the model (see figures presented in S1 File). On this basis, we decided that the analysis would use a parsimonious specification of the 'count' part of the model.

To account for expected curvilinearity, additional quadratic terms have been used in the case of four variables: ‘Geographical distance’, ‘Number of papers at destination’, ‘LinesXstop’, and ‘SeatsXstop’. We assume that the impact of enumerated variables on scientific collaboration is not uniformed across their possible values. In particular, the impact can be more pronounced at low values and gradually less distinct at high values (diminishing returns pattern). For example, we can expect that the difference between one and two direct flights between the same two cities should have substantial impact on the likelihood of research collaboration, while the difference between 11 and 12 direct flights can have less pronounced effect.

There is a lot of debate about the exponent characterizing the distance decay in the gravity model [75]. Using a quadratic term can not only be arbitrary but also lead to model misspecifications. Therefore, various values of the exponent used to model geographical distance were tested. Exponent values from 1.1 to 3, in increments of 0.1, were applied (for the details please refer to the S1 File). The result of exercise showed that different variants of the exponent do not translate into significant differences in the degree of the fit of the model. The extreme values of AIC and BIC differ in a negligible way. Coefficients of key variables, i.e., those measuring air transport connectivity and accessibility, are stable across compared models. Given the lack of substantial differences in the results generated by tested specifications, it was decided to use exponent 2 (‘Geographical distance squared’) in the main analyzes. This solution ensures consistency of the specifications presented in the article (squared terms used in other variables).

Because air transport makes little sense for very short distances and research scholars typically do not use private jets to get to nearby institutions, we excluded observations in which geodistance variable was less than 100 miles. The exclusion is specifically reasonable in the case of our research sample because the area within a 100-mile radius of three out four purposively selected ego-institutions (ASU, IUB, and IUPUI) was clearly within the catchment area of a single airport [76]. Thus in these cases, it was not possible to travel between an ego and its alters by scheduled commercial flights. UMICH constitutes a slightly different example. In the area within a 100-mile radius from UMICH’s campus in Ann Arbor there are also other airports with scheduled commercial flights apart from Detroit Metropolitan Airport. These are namely airports in Lansing, Flint, Kalamazoo, and Toledo. However, we assumed that it is very unlikely that travelers from Ann Arbor would opt for flying from DTW to one of those cites as air travel is less attractive than road transport, both in terms of cost and time (taking into account time needed to reach the airport in Detroit, located circa 25 miles from Ann Arbor, check-in and security, and than the time needed for living the airport and reaching the journey's end). In total, 55 observations were omitted, of which 4 for ASU, 23 for IUB, 22 for IUPUI, and 24 for UMICH. As a result, a restricted dataset used as a basis for estimations consisted of 8,925 observations, multidimensional links (co-authorships, geographical distance, air links, etc.) between four universities and theirs possible research collaborators. Sub-datasets for individual universities were as follows: ASU—2,241 observations, IUB—2,222, IUPUI—2,223, and UMICH—2,221.

We used Zero-inflated Negative Binomial Regression (ZINBR) model implemented in STATA [77]. However, we tested other models for count data: Poisson (PRM), Zero-Inflated Poisson (ZIP), and Negative Binomial Regression Model (NBRM). The results of estimation strongly suggest that ZINBR fits our data significantly better than PRM, ZIP, and NBRM.

The results section of the paper presents model specifications grouped into four tables. Specifications differ in terms of employed independent variables, as well as observations taken into account. Models from (1) to (14) are based on the full dataset, while models (15)-(34) are based on institutional sub-datasets. Model (1) is a reference model that includes only control variables and any of the air transport variables. Other models include various configurations of air transport accessibility and connectivity variables. The comparison of complete and restricted specifications allows for insights into complex relationships between scientific collaboration, air transportation, and geographic separation.

## Results

Table 4 presents estimation results of models with air transport connectivity and accessibility (models 6–9), as well as models without airport accessibility variable (2)-(5), compared to the reference model that does not include any transport variables (1). As expected, the basic model (1) with no air transport availability variables does significantly worse than all other models with transport variables included. This is evidenced by the fact that model (1) has the highest values of Akaike Information Criterion (AIC) and Bayesian information criterion (BIC). The difference in AIC and BIC between the model (1) and the second worst specification, model (2), highly exceeds 10 and can, therefore, be considered significant [78, 79]. The addition of air connectivity variables (models 2–5) noticeably improves the fit of the model (significant decrease in both AIC and BIC). Moreover, enriching the model with a variable describing the accessibility of the nearest airport (models 6–9) improves the fit even more. Consequently, models combining air transport connectivity and accessibility (6)-(9) perform significantly better than specifications comprising only connectivity variables (1)-(5). These results plainly indicate that not only the physical distance influences the intensity of scientific collaboration, but also, the actual transport accessibility plays a significant role.

**Table 4:** Research collaboration and air transport connectivity and accessibility.

Dependent variable: Number of co-authored papers	(1)	(2)	(3)	(4)	(5)	(6)	(7)	(8)	(9)
**Count part**									
Geographical distance (thous mi)	-0.364***	-0.315***	-0.247***	-0.271***	-0.292***	-0.292***	-0.218***	-0.246***	-0.269***
Geographical distance squared (thous mi)	0.019***	0.015***	0.011***	0.013***	0.015***	0.012***	0.008**	0.010***	0.012***
Number of papers at destination	0.116***	0.112***	0.107***	0.106***	0.105***	0.106***	0.100***	0.098***	0.098***
Number of papers at destination squared	-0.000***	-0.000***	-0.000***	-0.000***	-0.000***	-0.000***	-0.000***	-0.000***	-0.000***
Disciplinary similarity	0.025***	0.025***	0.023***	0.021***	0.021***	0.023***	0.021***	0.019***	0.019***
Disciplinary similarity squared	-0.000***	-0.000***	-0.000**	-0.000*	-0.000*	-0.000**	-0.000**	-0.000*	-0.000*
lines0stop		0.298***				0.334***			
lines0stop squared		-0.023***				-0.026***			
lines1stop			0.073***				0.078***		
lines1stop squared			-0.001***				-0.001***		
lines2stop				0.028***				0.029***	
lines2stop squared				-0.000***				-0.000***	
lines3stop					0.005***				0.005***
lines3stop squared					-0.000***				-0.000***
Distance to airport at destination (mi)						-0.011***	-0.012***	-0.012***	-0.012***
Constant	0.996***	0.881***	0.547***	0.459***	0.442***	1.178***	0.835***	0.750***	0.732***
**Inflate part**									
Number of papers at destination	-4.308***	-4.210***	-4.171***	-4.174***	-4.165***	-3.977***	-3.916***	-3.918***	-3.912***
Constant	-0.107	-0.104	-0.126	-0.14	-0.145*	-0.194**	-0.224**	-0.238**	-0.244**
Constant lnalpha	0.803***	0.791***	0.773***	0.772***	0.771***	0.776***	0.753***	0.753***	0.751***
**Statistics**									
Observations	8925	8925	8925	8925	8925	8925	8925	8925	8925
AIC	40808.4	40762.0	40629.6	40613.0	40600.7	40617.8	40459.7	40446.8	40433.4
BIC	40879.3	40847.2	40714.8	40698.2	40685.9	40710.0	40551.9	40539.1	40525.7
Cox-Snell R2	0.478	0.499	0.488	0.489	0.490	0.489	0.498	0.499	0.499
Cragg-Uhler/Nagelkerke R2	0.480	0.502	0.491	0.492	0.493	0.492	0.501	0.501	0.502

Significance levels:

* p<0.05;

** p<0.01;

*** p<0.001.

Not only the existence of flight connection matters, but also its passenger capacity. Taking into account the number of available seats improves model’s fit as measured by AIC and BIC. This is visible by comparing models based on simple connectivity variable, ‘LinesXstop’ (Table 4), and models based on seats-weighted connectivity variable, ‘SeatsXstop’ (Table 5). In the case of specifications with direct connections (models 6 and 10), connections up to one stop (models 7 and 11), and connections up to two stops (models 8 and 12), BIC and AIC statistics are in favor of seats-weighted connectivity variable. However, in the case of connections up to three stops, non-weighted connectivity variable does better. This is probably because connections requiring up to three changes are rare, so in their case, the most important thing is the existence of a connection, not its capacity. Regardless, in the group of models presented in Tables 4 and 5, model (12), involving seats-weighted connections up to two stops, has the lowest AIC and BIC values, and therefore it can be preferred as best suited to the analyzed data.

**Table 5:** Research collaboration and air transport–seats capacity.

Dependent variable: Number of co-authored papers	(10)	(11)	(12)	(13)
**Count part**				
Geographical distance (thous mi)	-0.292***	-0.262***	-0.285***	-0.301***
Geographical distance squared (thous mi)	0.012***	0.011***	0.012***	0.013***
Number of papers at destination	0.106***	0.100***	0.098***	0.101***
Number of papers at destination squared	-0.000***	-0.000***	-0.000***	-0.000***
Disciplinary similarity	0.023***	0.021***	0.019***	0.019***
Disciplinary similarity squared	-0.000**	-0.000*	-0.000*	-0.000*
Seats0stop	2.631***			
Seats0stop squared	-1.602***			
Seats1stop		0.465***		
Seats1stop squared		-0.045***		
Seats2stop			0.182***	
Seats2stop squared			-0.006***	
Seats3stop				0.003***
Seats3stop squared				-0.000***
Distance to airport at destination (mi)	-0.011***	-0.012***	-0.012***	-0.012***
Constant	1.181***	0.970***	0.877***	1.086***
**Inflate part**				
Number of papers at destination	-3.967***	-3.925***	-3.926***	-3.994***
Constant	-0.193**	-0.225**	-0.245**	-0.230**
Constant lnalpha	0.775***	0.752***	0.749***	0.760***
**Statistics**				
Observations	8925	8925	8925	8925
AIC	40613.5	40448.5	40415.0	40475.9
BIC	40705.8	40540.8	40507.2	40568.2
Cox-Snell R2	0.489	0.499	0.501	0.497
Cragg-Uhler/Nagelkerke R2	0.492	0.501	0.504	0.500

To ensure meaningful coefficients SeatsXstop variable is divided by 1000.

Significance levels:

* p<0.05;

** p<0.01;

*** p<0.001.

Based on AIC and BIC, we know which of the compared models has better performance. However, these statistics do not allow assessing the overall fit of the model to the data. This task is complex for ZINBR models because one cannot use a simple r-square measure of fit [80, 81]. Therefore, two pseudo r-square measures have been used: Cox-Snell pseudo r-square and Cragg-Uhler/Nagelkerke pseudo r-square (as defined in 77). These measures are constructed in such a way that their interpretation is similar to r-square. On this basis, it can be concluded that all the analyzed models have at least a satisfactory fit to the data. For models based on the Cox-Snell full dataset, the pseudo r-square values range from 0.478 to 0.501, and Cragg-Uhler/Nagelkerke the pseudo r-square values range from 0.480 to 0.504. In the case of models based on institutional subsets, it is evident that the subset for UMICH has a slightly better fit (psuedo r-squares in the range of 0.602–0.607) than subsets of the other three institutions (psuedo r-squares in the range of 0.466–0.504). Furthermore, a prediction experiment was performed to assess the fit of the models. For this purpose, the data set has been randomly divided into two parts—a training set of 80% of observations and a test set of 20% of observations. The training set was passed through all the model specifications discussed in the article. Then, based on the data from the test set, the predicted values of the outcome variable, i.e. the number of co-authored papers, were calculated. In the next step, the predicted value of the outcome variable was compared to the actual value from the test set. This was done using a simple linear model (OLS) in which the left side of the equation is the actual number of co-authored papers, and the right side of the equation is the number of co-authored papers predicted. The use of OLS enables the calculation of r-square–a well-known and easy to interpret measure of fit. The results of this exercise testify to the relatively good fit of the model to the data: the predicted values of the number of co-authored articles explain about half the variability of the actual number of co-authored articles (for the details please refer to the S1 File).

Further analysis of the compared models reveals, firstly, that direct connections have a stronger impact on the probability of scientific cooperation than flights requiring transfers—see specifications (14)-(18) with dummy variables for direct and connecting flights presented in Table 6. In the case of destinations that have no direct flight connection and requires minimum one stop, the number of expected co-publication decreases by a factor of 0.5 as compared to destinations that can be reached with a single flight (for a full dataset as specified by model 14). Secondly, the greater the number of transfers required, the weaker the effect on the dependent variable. This is evidenced by the fact that the models with only direct flights—specifications (2), (6), and (10)—have the highest coefficient of air transport variable (Lines0stop and Seats0Stop). In turn, models with up to one, two or three stops show decreasing values of air transport coefficient (Lines1stop and Seats1stop, Lines2stop and Seats2stop, Lines3stop and Seats3stop, respectively). This result is in line with expectations. Direct flights and those requiring fewer transfers are more convenient for passengers than connections requiring many stops. At the same time, not only air transport connectivity matters but also the distance between the location of the co-authors and their nearest airport. The results of the estimation confirm the common sense of expectations that the proximity of the airport is advantageous, at least in the case of long-distance cooperation, which from time to time requires air travel.

**Table 6:** Research collaboration and air transport–Direct and connecting flights.

Dependent variable:	Full dataset	ASU	IUB	IUPUI	UMICH
Number of co-authored papers	(14)	(15)	(16)	(17)	(18)
**Count part**					
Geographical distance (thous mi)	-0.143***	-0.449***	-0.256***	-0.407***	-0.104*
Geographical distance squared (thous mi)	0.002	0.025***	0.013*	0.031***	-0.003
Number of papers at destination	0.102***	0.104***	0.102***	0.087***	0.132***
Number of papers at destination squared	-0.000***	-0.000***	-0.000***	-0.000***	-0.001***
Disciplinary similarity	0.018***	0.051***	-0.060***	0.021**	0.044***
Disciplinary similarity squared	0	-0.000***	0.001***	0	-0.000***
Minimum number of stops to reach destination (compared to direct flight):					
1 stop	-0.694***	-0.272*	-0.374	-1.089***	-0.393***
2 stops	-1.263***	-0.277	-0.824**	-1.309***	-0.594***
3 stops	-1.554***	-1.246***	-1.107***	-1.296***	-0.462
Distance to airport at destination (mi)	-0.012***	-0.011***	-0.010***	-0.009***	-0.013***
Constant	1.868***	0.900*	3.643***	2.146***	1.229***
**Inflate part**					
Number of papers at destination	-4.025***	-3.560***	-4.882***	-2.073***	0.034
Constant	-0.218**	0.001	0.718***	0.364**	-25.309
Constant lnalpha	0.744***	0.543***	0.633***	0.621***	0.655***
**Statistics**					
Observations	8907	2241	2222	2223	2221
AIC	40356.6	9478	8441.1	8061.3	13486.1
BIC	40455.9	9558	8521.0	8141.2	13566.0
Cox-Snell R2	0.501	0.500	0.471	0.479	0.602
Cragg-Uhler/Nagelkerke R2	0.504	0.504	0.477	0.485	0.602

Significance levels:

* p<0.05;

** p<0.01;

*** p<0.001.

The relationship between air connectivity and the number of co-authored papers is not linear. All the squared air connectivity variables are significant in specifications (1)-(13). Negative coefficients of the quadratic terms suggest that at some point, the connectivity is so high that its further increase (e.g. adding one more flight between given airports) has far less impact on collaboration than the similar increase at low levels of the overall connectivity.

The number of scientific papers affiliated in potentially cooperating destinations serves two functions in presented models: first, as specified in the inflate part, and second, as specified in the count part. The count part can be interpreted similarly to standard maximum likelihood models. Firstly, the increase in the number of articles at destination translates into reduction in the likelihood of a complete absence of co-authored articles. In other words, an increase in the number of articles at destination decreases the likelihood that the variable ‘number of co-authored articles’ will equal zero. Secondly, as the count part of the models shows, the more articles in the cooperating destination, the higher the expected number of co-authored papers between the ego and the destination. However, this relationship is more complex, as indicated by the significant quadratic term for the number of articles at the destination. Negative coefficients of the quadratic term indicate the curvilinear shape of the relationship: as the number of articles increases, its positive influence on the number of co-authored articles is flattening out.

In all presented models, geographical distance is negatively associated with research collaboration. The higher the distance, the smaller the number of co-publications. Furthermore, the effect is also curvilinear. In this case, positive coefficient of the squared variable suggests that the negative influence of physical distance on collaboration decreases gradually as the geographic separation increases. This can be interpreted as follows: the difference between, for example, 9,100 or 9,200 miles does not translate into a significant difference for the person considering a trip to such a remote place. But the difference between 100 and 200 miles means, approximately, a two-fold lengthening of the journey and thus, can be a significant factor influencing the decision.

**Fig 3:**
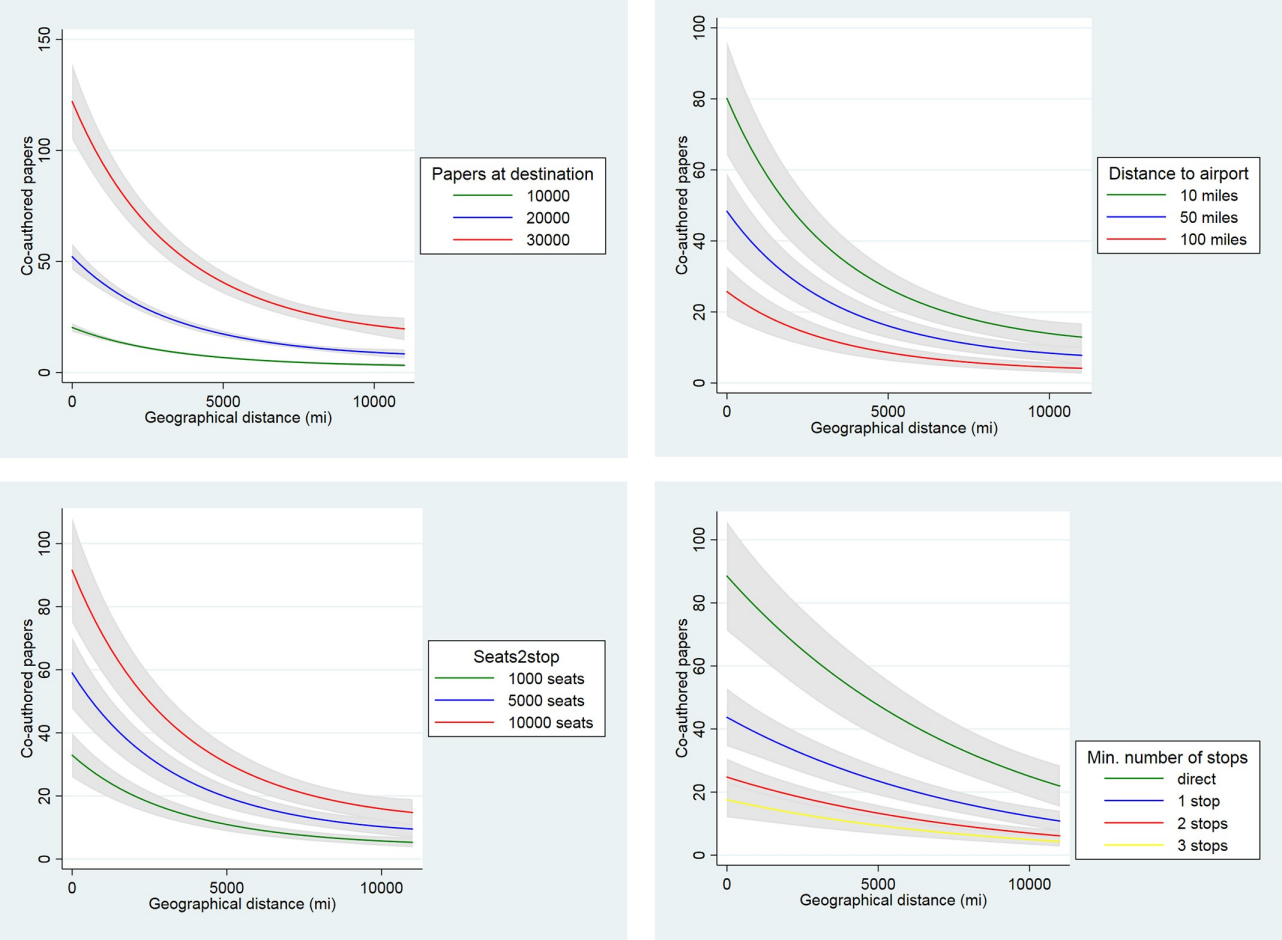
Predicted number of collaborative papers at different values of selected independent variables. The estimations are based on model (12) in the case of ‘Papers at destination’, Searts2stop’, and ‘Distance to airport’. For ‘Minimum number of stops’ model 14 has been employed.

The influence of geographical distance on the number of co-publications is modified by air transport connectivity and accessibility, as well as by scientific capacity of collaborators. The low number of papers at the destination, less than one thousand, usually translates into the low number of co-publications, no matter the distance. On the other hand, for destinations that accumulated high research capacity, the distance matters a lot. For example, in the case of destinations with 30 thousand papers, the decrease in the distance from 4,000 to 1,000 miles raises the expected number of co-publications twice, from circa 50 to 100. While the decrease from 10,000 to 7,000 miles (i.e., by the same number of miles, 3,000), raises the expected number of co-publications by no more than ten papers. Similarly, for the low values of connectivity and accessibility, the relation between geographical distance and expected number of co-authrships is flatter than for high values of those variables. Furthermore, the distance matters significantly more in the case of direct flights, than for connections requiring one, and in particular, two or three transfers (see Fig 3). This is reasonable as direct flights are constrained by technical capacities of aircrafts, as well as regulatory requirements, in particular limits for flight duty periods for crew member’s [82].

Estimations based on institutional sub-datasets (Tables 6 and 7) show that relationship between air transport connectivity and research collaboration is not homogeneous across the four universities. Nevertheless, in each of the analyzed cases, air transport connectivity explains some significant part of the variation of co-publications. The differences relate primarily to the importance of direct and connecting flights. The comparison of IUB and IUPUI is particularly interesting. Both institutions are served by the same airport. Thus they have the same air transport connectivity (However, it should be emphasized that IUB is located at a much greater distance to the Indianapolis airport than IUPUI). In the case of IUPUI, direct flights are the most significant predictors of co-publications, both statistically and substantially. While for IUB the availability of direct flights is not essential, but connections up to one and two stops matters much more than for IUPUI—compare specifications (23)-(30). Such divergent patterns can be possibly attributed to organization-specific research collaboration networks, related to the disciplinary composition of institutions. The biggest difference between IUPUI and IUB is that the former hosts the School of Medicine, while the latter does not. This institutional specificity is clearly visible in the disciplinary composition of research outputs. In the case of IUPUI, the three top disciplines of articles published in years 2008–2013 are: medical specialties (35,9%), health professionals (17,1%), and brain research (13,3%). For IUB the top tree disciplines are: social sciences (21%), math & physics (15,3%), and brain research (8,6%). (The detailed information on the disciplines of articles published by the case study institutions is provided in the S1 File). Moreover, disciplinary similarity plays a different role for IBU than the other three institutions. For these latter, the expected number of co-authored articles increases as disciplinary similarity increases. For IUB, the expected number of co-authored articles is higher when dyscylinary similarity is smaller. Here again, the explanation may be that IUB, not having a medical school, is looking for complementarity in cooperation with institutions with developed medical research. The observed heterogeneity suggest that the further research should scrutinize institutional differences related to their disciplinary specializations.

**Table 7:** Research collaboration and air transport—institutional sub-datasets.

Dependent variable: Number of co-authored papers	ASU				IUB				IUPUI				UMICH			
	(19)	(20)	(21)	(22)	(23)	(24)	(25)	(26)	(27)	(28)	(29)	(30)	(31)	(32)	(33)	(34)
**Count part**																
Geographical distance (thous mi)	-0.452***	-0.459***	-0.448***	-0.481***	-0.374***	-0.324***	-0.355***	-0.375***	-0.484***	-0.437***	-0.466***	-0.516***	-0.181***	-0.167***	-0.172***	-0.185***
Geographical distance squared (thous mi)	0.025***	0.026***	0.024***	0.027***	0.021***	0.018**	0.019***	0.021***	0.037***	0.035***	0.037***	0.040***	0.003	0.002	0.002	0.003
Number of papers at destination	0.104***	0.103***	0.101***	0.104***	0.103***	0.100***	0.097***	0.100***	0.088***	0.085***	0.083***	0.088***	0.130***	0.130***	0.129***	0.131***
Number of papers at destination squared	-0.000***	-0.000***	-0.000***	-0.000***	-0.000***	-0.000***	-0.000***	-0.000***	-0.000***	-0.000***	-0.000***	-0.000***	-0.001***	-0.001***	-0.001***	-0.001***
Disciplinary similarity	0.052***	0.052***	0.049***	0.053***	-0.066***	-0.062***	-0.063***	-0.062***	0.021**	0.020**	0.019**	0.018**	0.043***	0.044***	0.042***	0.044***
Disciplinary similarity squared	-0.000***	-0.000***	-0.000***	-0.000***	0.001***	0.001***	0.001***	0.001***	0	0	0	0	-0.000***	-0.000***	-0.000***	-0.000***
Seats0stop	1.099*				2.254				5.853***				1.700*			
Seats0stop squared	-0.531				-2.009				-5.671**				-1.387*			
Seats1stop		0.113				0.423***				0.362**				0.282***		
Seats1stop squared		-0.007				-0.069*				-0.029				-0.027**		
Seats2stop			0.082**				0.156***				0.150***				0.121***	
Seats2stop squared			-0.003*				-0.008*				-0.006				-0.004**	
Seats3stop				0				0.002**				0.001				0.002***
Seats3stop squared				0				-0.000*				0				-0.000**
Distance to airport at destination (mi)	-0.011***	-0.011***	-0.011***	-0.011***	-0.009***	-0.010***	-0.010***	-0.010***	-0.008***	-0.008***	-0.008***	-0.008***	-0.013***	-0.013***	-0.013***	-0.013***
Constant	0.606	0.583	0.509	0.621	3.496***	3.132***	3.090***	3.280***	1.101***	0.866***	0.784***	1.142***	1.023***	0.810***	0.714***	0.841***
**Inflate part**																
Number of papers at destination	-3.530***	-3.505***	-3.502***	-3.524***	-4.863***	-4.864***	-4.838***	-4.847***	-1.998***	-2.062***	-2.046***	-2.137***	-6.602	0.029	0.028	0.019
Constant	0.015	0.007	-0.004	0.01	0.753***	0.722***	0.710***	0.723***	0.380**	0.356**	0.339**	0.371**	-1.900***	-19.674	-20.338	-19.224
Constant lnalpha	0.543***	0.545***	0.542***	0.547***	0.638***	0.635***	0.631***	0.639***	0.621***	0.634***	0.634***	0.652***	0.595***	0.646***	0.642***	0.652***
**Statistics**																
Observations	2244	2244	2244	2244	2228	2228	2228	2228	2229	2229	2229	2229	2224	2224	2224	2224
AIC	9488.9	9489.1	9481.5	9492.5	8473.2	8456.9	8451.1	8462.5	8083.1	8083.8	8080.6	8100.1	13497.1	13481.0	13471.8	13491.9
BIC	9563.2	9563.4	9555.8	9566.8	8547.4	8531.1	8525.3	8536.7	8157.4	8158.0	8154.8	8174.3	13571.3	13555.2	13546.0	13566.1
Cox-Snell R2	0.499	0.499	0.500	0.498	0.466	0.470	0.471	0.468	0.476	0.475	0.476	0.472	0.602	0.605	0.607	0.603
Cragg-Uhler/Nagelkerke R2	0.503	0.502	0.504	0.502	0.472	0.475	0.477	0.474	0.482	0.482	0.483	0.478	0.603	0.606	0.607	0.604

To ensure meaningful coefficients SeatsXstop variable is divided by 1000.

Significance levels:

* p<0.05;

** p<0.01;

*** p<0.001.

## Discussion and conclusions

The paper makes two contributions. First, we show that air transport availability is an important factor for scientific collaboration, even when controlling for geographical distance and research capacities of collaborators. Second, both air transport connectivity (direct and indirect air connections between airports) and accessibility (distance to the nearest airport) are important correlates of scientific collaboration. Presented estimation results provide evidence that more flight connections and greater seat capacity correlates with the increased number of co-publications. Also, proximity of airport at collaborating destination is positively related to the expected number of co-authored papers. Moreover, direct flights and flights with one transfer are more valuable for intensifying scientific collaboration than travels involving more connecting flights. One additional direct flight rise the expected number of co-publications by a factor of 1.40, while additional connection requiring up to two stops rises the number by a factor of 1.03. The results of our study are in line with conclusions from broader research corpus highlighting the importance of air transport for the economic development of cities and regions [83]. In particular, the availability of direct flights is seen as a significant predictor of a city’s fortunes [82].

Estimations based on four separate institutional sub-datasets show that the relationship between transport accessibility and scientific cooperation is not uniform. For some institutions—Indiana University-Purdue University Indianapolis in the first place—direct flights are more valuable predictors of distant co-publications, while for other three institutions indirect connections up to one or two stops better explain their collaboration patterns. This diversity can be related to different research profiles of studied universities—however the phenomena needs further investigation that goes beyond the scope of this study. Not only research organizations differ in scientific specialization, but also scientific disciplines are spatially biased regarding propensity to collaborate [37, 84]. For example, collaboration in experimental particle physics is far more spatially bound than collaboration in theoretical mathematics. This organizational and disciplinary diversity shapes spatial patterns of collaboration, in a dynamic coopetitive—i.e., simultaneously cooperative and competitive—processes [85].

Two limitations of the presented approach have to be underlined. First, the direction of the relationship between air transport availability and research collaboration is ambiguous. Increasing collaboration can be both the result and the cause of transport availability. Development of collaborative relations between distant locations indeed rises the demand for transport. However, based on the results of a quasi-experimental study by Catalini, Fons-Rosen and Gaulé [55], we can expect that causal relation from transport connectivity to scientific collaboration also happen. Moreover, the circular cumulative causation can be expected—more collaboration leads to higher transport demand and in result greater transport capacity, which in turn induces more collaboration, and so forth. The second limitation is related to the dataset used in this study. We focused on four non-randomly selected universities located in the US. As such, our results cannot be extended to the entire population of universities/cities. In other socio-economic and geographical contexts, the role of air transport can be different. For example, in Europe, Japan, and increasingly in China, railway connectivity can be more critical than air transport, at least up to some geographical distance.

Future studies might use the method presented here and apply it to the total set of all research universities and their geolocations. Secondly, they might control for more covariates, e.g., citation data, to capture other important factors such as the impact of research reputation on collaboration. Thirdly, other modes and measures of research collaboration should be examined, e.g., co-inventorship via patent or co-investigatorship via funding data. Fourth, different modes of transport should be incorporated: road and railroad connectivity between spatial units in question, as well as various modes of access to airports. We expect that essential insights can be gained by combining multimodal transport connectivity and multimodal research collaborations, comparing and integrating co-publications, co-patenting, and collaborative research projects. Fifth, subsequent studies of the discussed topic should employ experimental or quasi-experimental research designs to establish robust causal claims [86].

In conclusion, it is worth emphasizing that the relationship between air transport availability and scientific collaboration does not provide sufficient basis to formulate a straightforward policy recommendation indicating that more flights are necessary to boost scientific collaboration. The major concern is the adverse environmental impact of air travel [87], especially in the context of the academic hypermobility culture [88]. Another, no less important, problem relates to the financial and social burdens of academic travel that undermines diversity and equity of research community across countries, organizations, and demographics [83, 89, 90]. Finally, the COVID-19 epidemic has highlighted the importance of ways of scientific exchange that does not require travel and physical presence in one place. There are many urgent questions in this context. To what extent can virtual meetings and conferences replace travel and personal meetings [91–93]? Under what conditions? What will be the impact of reducing academic mobility on the development of science [94]? How will these changes affect power and prestige in the global science system [95, 96]?

## Supporting information

S1 File (DOCX)

S1 Fig (JPG)
