# Supplementary material for: The impact of air transport availability on research collaboration: A case study of four universities
Source: PLoS One. 2020 Sep 4;15(9):e0238360. doi: 10.1371/journal.pone.0238360 (PMC7473522; doi:10.1371/journal.pone.0238360)
Supplement: S1 File — (DOCX) [file pone.0238360.s001.docx]

**Supporting information for:**

**The impact of air transport availability on research collaboration: A case study of four universities**
Adam Ploszaj^1^, Xiaoran Yan^2^, Katy Börner^2,3^

^1^ Centre for European Regional and Local Studies EUROREG, University of Warsaw, Warsaw, Poland

^2^ Indiana Network Science Institute, Indiana University, Bloomington, Indiana, United States of America

^3^ School of Informatics, Computing, and Engineering, Indiana University, Bloomington, Indiana, United States of America

Contents

[1. Data Acquisition and Preparation 1](#_Toc43074431)

[1.1. WoS Publication Data 1](#_Toc43074432)

[1.2. Flight Traffic Data 5](#_Toc43074433)

[2. Distributions of variables 8](#_Toc43074434)

[3. Model specification and robustness checks 10](#_Toc43074435)

[4. Prediction experiment 15](#_Toc43074436)

[5. Expertise Profiles: Science Maps 24](#_Toc43074437)

[6. Code instructions for result replication 27](#_Toc43074439)

# Data Acquisition and Preparation

Two different types of data are used in this analysis: WoS publication data and flight traffic data. The acquisition, preprocessing, and matching of these datasets is detailed subsequently.

## WoS Publication Data

The IU Network Science Institute (IUNI) Clarivate Analytics (previously Thomson Reuters)’ Web of Science dataset (1900-2013)^[[1]](#footnote-1)^ is used in this study. The data comprises publications from

- Science Citation Index Expanded
- Social Sciences Citation Index
- Arts &Humanities Citation Index
- Book Citation Index -- Science
- Book Citation Index -- Social Sciences & Humanities
- Conference Proceedings Citation Index -- Science & Technical

We define a relevant author as someone who has an affiliated address that contains the case insensitive search terms “Indiana Univ”, “Univ Michigan” or “Arizona State Univ”. There are 121,956 papers published in WoS that have at least one author with at least one affiliation at one of these three institutions. Only 4,995 papers associated with the three institutions are published before 2007, see Fig. 1. Therefore, we restrict our analysis to the years 2008-2013, leaving 116,961 papers in the dataset.

**Figure 1:** The histogram of paper counts per publication year

From the 111,961 papers published during 2008-2013, we discard 20,741 papers that have no affiliations. Next, we examined the number of papers per “document type” (“doctype” column), see histogram in Figure 2.

The “article” document type is the most widely used, we restrict the data to this subset of 74,211 out of 91,220 publications. We further restricted the set of papers to those with complete affiliation and deleted 10,590 papers with incomplete affiliations for all authors, leaving 63,621 total publications for this study.

**Figure 2:** The histogram of document types

For papers with more than 30 authors (355 out of 63,621 total papers), the first and last 15 authors were kept. For the analysis of collaborations, we only keep the first three addresses for authors with more affiliations (2,637 out of 344,665 total paper-author pairs were affected). The decision here is to maintain a manageable size of collaborations that is also meaningful. The number of authors per paper and number of affiliations per author is shown in Figure 3.

**Figure 3:** Histogram of author counts per paper (left) and affiliation counts per author (right)

We choose to map addresses to city-level geolocations. We extracted city, state, and country combinations from the “city+state+country” tags provided by WoS. The Bing Geocoder in the Science of Science (Sci2) Tool was used to geocode the cities, following the workflow detailed in Section 4.7.3 of the Sci2 Wiki.^[[2]](#footnote-2)^ With additional manual corrections for all geo-tags with more than 20 appearances as affiliations, all 6,874 unique “city+state+country” tags were successfully geo-located. The table is stored as “[cityListJoin.csv](https://github.com/everyxs/FlightCoauthor/blob/master/data/WoS/cityListJoin.csv)”, with following columns:

- Row id, full address, city, state, country, latitude, longitude, metro join id, standard name

To disambiguate city names, we aggregated cities into metro areas according to statistical areas in the US and EU (represented by the “metro join id” column). We manually added metro area mappings for major cities in other countries including Canada, China, India, Japan, etc. The resulting merge table is called “[cityListMerged.csv](https://github.com/everyxs/FlightCoauthor/blob/master/data/WoS/cityListMerged.csv)”, with following columns:

- Full address, latitude, longitude, standard name, country, number of affiliations, merged row id list

Cities from the previous table are merged into bigger metro/statistical areas. The mapping is given in the rowIDlist column. A total of 3,699 unique “city+state+country” strings are merged into other metro arears, resulting in 3,175 basic level geo-locations, as illustrated in Figure 4.


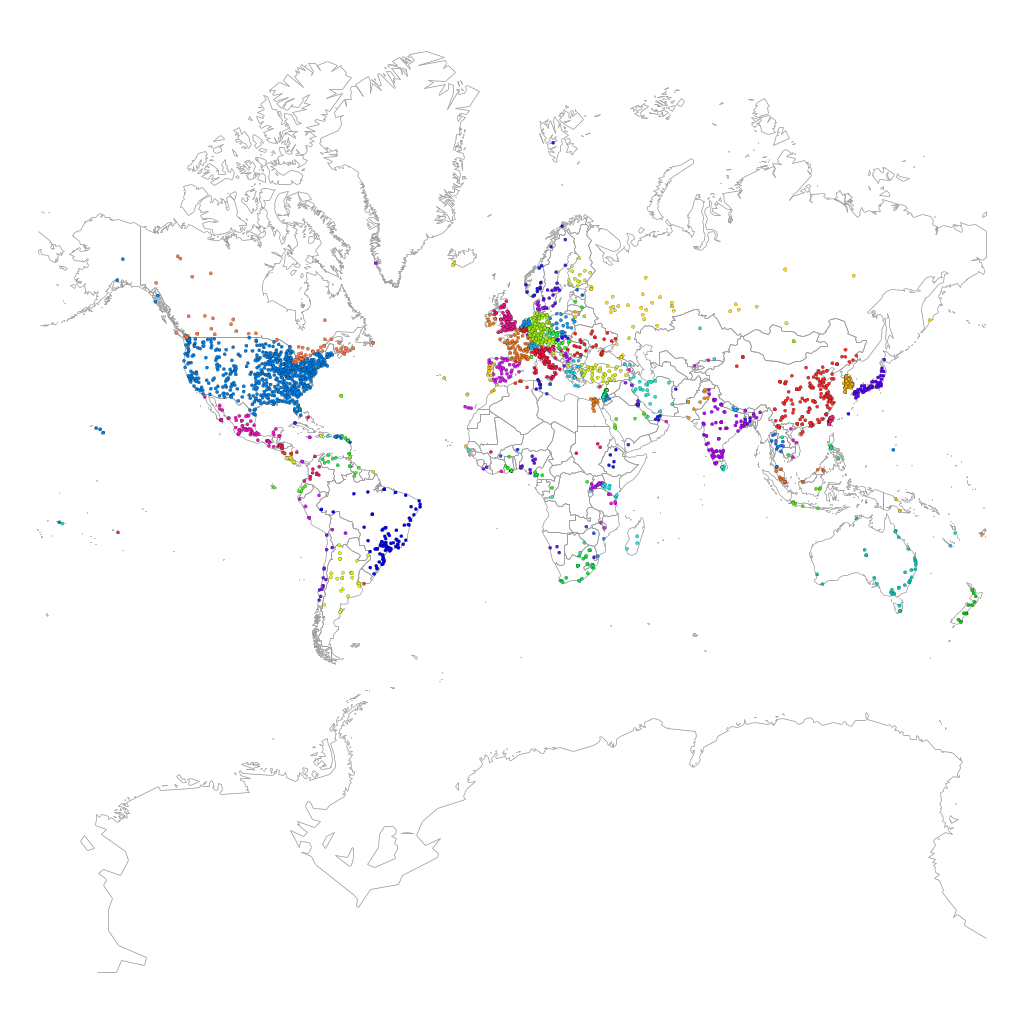


**Figure 4:** Merged cities and metro areas on the world map under this study.

Using the aforementioned geo-mapping, we can constructed co-authorship relationships between cities/metros. In this study, we focus on four university campuses and their ego-centric co-locations, namely, Arizona State University at Tempe (ASU), Indiana University Bloomington (IUB), Indiana University-Purdue University Indianapolis (IUPUI) and University of Michigan at Ann Arbor (UMICH).

To account for each city/metro’s background overall academic activity, we introduce an external data set of academic productivity (Mazloumian, Helbing, Lozano, Light & Börner, 2013), stored in the table “[locations.csv](https://github.com/everyxs/FlightCoauthor/blob/master/data/WoS/locations.csv)”, with following columns:

- Location id, location name, longitude, latitude, sum of papers, merged cities

Here the column “Sum of Papers” is used to capture the overall academic activity. We mapped the locations with at least five papers to the aforementioned city/metro level geolocations, merging locations that are within 10 kilometers.

To allocate the 63,621 publications to the four universities under investigation, we consider the following four cities/metros as the egos of the network analysis

- UMICH: Ann Arbor MI , United States
- ASU: Tempe AZ United States
- IUB: Bloomington, United States
- IUPUI: Indianapolis IN, United States

Each paper in our dataset that has at least one affiliation with any of the four city/metro will be included for the construction of the corresponding university-ego network. Notice that if there are other academic institutions in the aforementioned cities/metros they were merged with the ego.

## Flight Traffic Data

Global flight traffic data was downloaded from the web site OpenFlights^^[[3]](#footnote-3)^^. We used two data files, with the first file being “[airports.csv](https://github.com/everyxs/FlightCoauthor/blob/master/data/WoS/airports.csv)”, with following columns:

- Airport id, name, city, country, IATA/FFA code, ICAO code, latitude, longitude, altitude, timezone

The second file is “[routes.csv](https://github.com/everyxs/FlightCoauthor/blob/master/data/WoS/routes.csv)”, with following columns:

- IATA code of the operating Airline, airline ID, IATA code of the source airport, airport ID of the source (mapped to “airports.csv”), IATA code of the destination airport, airport ID of the destination (mapped to “airports.csv”), number of stops during the flight, IATA equipment code (plane model)

To get a more accurate measure of flight traffic, we translated the IATA equipment code to number of passenger seats in corresponding plane models, , captured by the table “[planeWake.csv](https://github.com/everyxs/FlightCoauthor/blob/master/data/WoS/planeWake.csv)”, with following columns:

- IATA equipment code, manufacturer, type/model, wake, seats

The table is based on the IATA wake table from internet^^[[4]](#footnote-4)^^, and we manually input the number of typical passenger seats for popular plane models from Airbus and Boeing. For the other models, we used a general estimation that maps Wake “L” to 40 seats, “M” to 120 seats and “H” to 360 seats. When a flight listed multiple plane models, we took the arithmetic average.

We constructed a weighted flight network for flight routes by joining the first two tables, and a second weighted flight traffic network by also including the plane ‘Wake’ column. After filtering out disconnected airports, the resulting two networks have 3,253 nodes and 37,133 directed weighted edges, see Figure 1. Note that rather small airports, e.g., Monroe County Airport near Bloomington, are not covered in the dataset. Another observation is that almost all routes are one-way flights with 0 stops, and the flight traffic between two airports is usually symmetrical.

Following the city to metro mapping we did for the universities, we merge airports in the same city and metro area into a single node. The table “[airports_AP_joined_new.csv](https://github.com/everyxs/FlightCoauthor/blob/master/data/WoS/airports_AP_joined_new.csv)” extends the original “[airports.csv](https://github.com/everyxs/FlightCoauthor/blob/master/data/WoS/airports.csv)” with metro mappings and is used as the input file. The resulting mapping of merged airports is stored in the table “[airportMergeMap.csv](https://github.com/everyxs/FlightCoauthor/blob/master/data/WoS/airportMergeMap.csv)”, with following columns：

- airport ID (mapped to “airports.csv”), name, metro join id (mapped to “cityListJoin.csv”), latitude, longitude, airport merge list (mapped to “airports.csv”)

Here, the airport merge is indicated by the “airport merge list” column, where the airport with the highest count of total seats is selected to represent all other airports in the same metro-area by combining all flights. Connecting the nodes with the merged airlines, we have a directed weighted network with 2,854 nodes and 33,113 edges. Another observation of the dataset is that almost all traffic flows are symmetric between airports. For computational simplicity, we thus treat the air traffic network as undirected. After filtering out disconnected airports, both the flight count and the seat number weighted networks have 2,854 nodes and 16,877 weighted edges, see Figure 5 and the [graphML](https://github.com/everyxs/FlightCoauthor/blob/master/data/WoS/airlinesMerged.graphml) file.


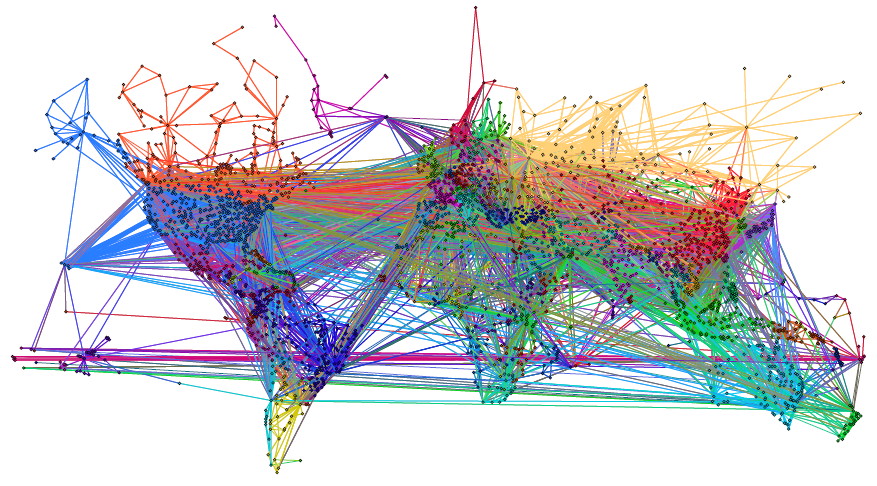


**Figure 5:** Air traffic data network (A) with merged cities and metro areas

In particular, the four egos are associated with the following three airports:

- UMICH: DTW (20.8 mi from Ann Arbor), 199 direct flights with 31384.2 seats
- ASU: PHX (4.3 mi from Tempe), 255 direct flights with 37238.5 seats
- IUB & IUPUI: IND (40.1 mi from IUB, 8.1mi from IUPUI), 62 direct flights with 7907.7 seats

To capture the total possible flight traffic flow from one city to another and up to four connecting flights, we aggregate all possible paths up to 3 stops. This is done by a weighted sum of powers of the direct flight adjacency matrix W:

$$F=W+ {[W}^{2}]^{\frac{1}{2}}+{[W}^{3}]^{\frac{1}{3}}+{[W}^{4}]^{\frac{1}{4}}$$

Notice here the flow $W^{x}(ij)$ represents the number of flights/seats from node *i* to node *j* by taking $x$ connecting flight(s), and each powered term of $W$ is normalized by taking an element-wise inverse power. Using this weighted power series, we calculated six total flight flow networks with 0, 1, 2 and 3 stops for both flight networks, four for flight route counts, and another four with the additional number of seats per flight information.

Next, we map each metro-level geolocations in a given ego-centric collaboration network with one of the 2,854 airport locations based on geo-distance, associating each affiliation with the closest airport. See Figure 6 for a visualization of the alignment.

**Figure 6.** Aligned collaboration ego-network (bottom) and the global airline network (top).

By mapping affiliations and airports to the same city/metro level geo-locations, we constructed the following data table “[SelectCities[Edges].csv](https://github.com/everyxs/FlightCoauthor/blob/master/data/WoS/SelectCities%5BEdges%5D-full.csv)” for our data modeling and analysis, with following columns:

- Source id, target id, edge id, dist2air, mass, seatsXstop, lineXstop, GeoDist, CollabPaper

Here, each row represents a pair of city/metro level geo-locations that have affiliated authors that collaborated on papers of the extracted WoS dataset. The “SeatsXStop” and “LinesXStop” columns are the eight aforementioned total flight flow networks. The “CollabPaper” column counts the number of articles that such collaboration has happened in our dataset.

Aggregating all four ego networks, we have a total of 10,060 alters. By examining the combinations of Dist2Air+MASS+geodist 1,080 duplicated ego-alter pairs were identified. Excluding these duplicates leads to a final datasets with 8,980 observations. For details of the variables and analysis, please refer to the main text of the paper.

The other columns are calculated according to the following “[SelectCities[Nodes].csv](https://github.com/everyxs/FlightCoauthor/blob/master/data/WoS/SelectCities%5BNodes%5D.csv)” table, with following columns:

- Node id, name, latitude, longitude, country, Dist2Air, mass

where node id is mapped with the “Source-Target” pairs in the edge table. The “Mass” column in the edge table is simply the product of the overall academic activities of the two end nodes and the “GeoDist” column is calculated according to the geo coordinates of the nodes.

# Distributions of variables

In this section, we present distributions of variables used in the study. In general, the variables are not distributed normally and some of them are highly skewed with an excessive number of zero-valued observations.


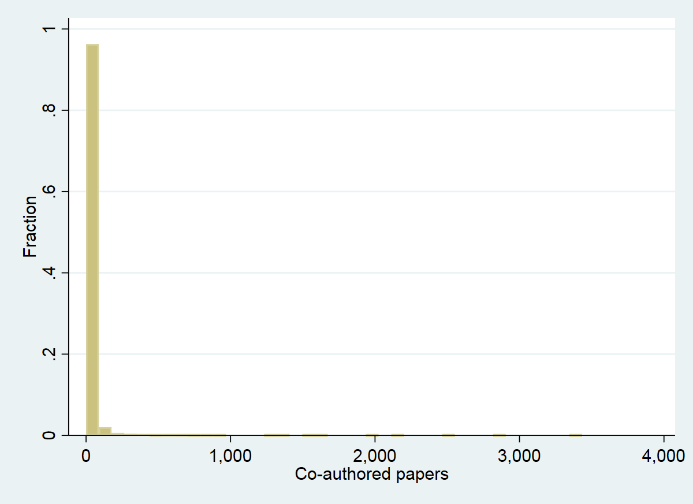

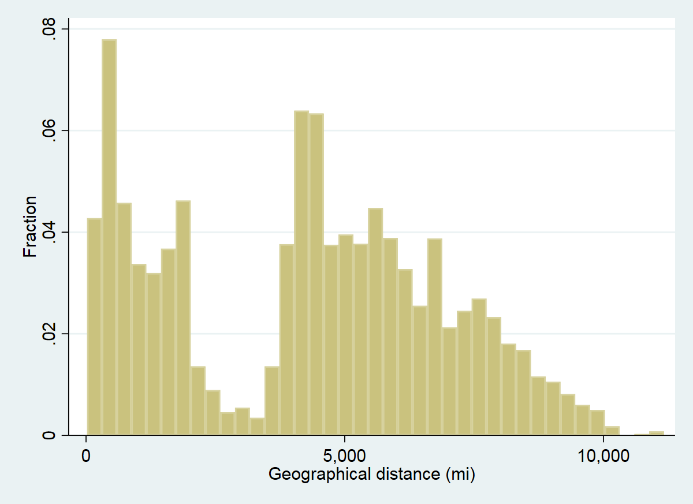


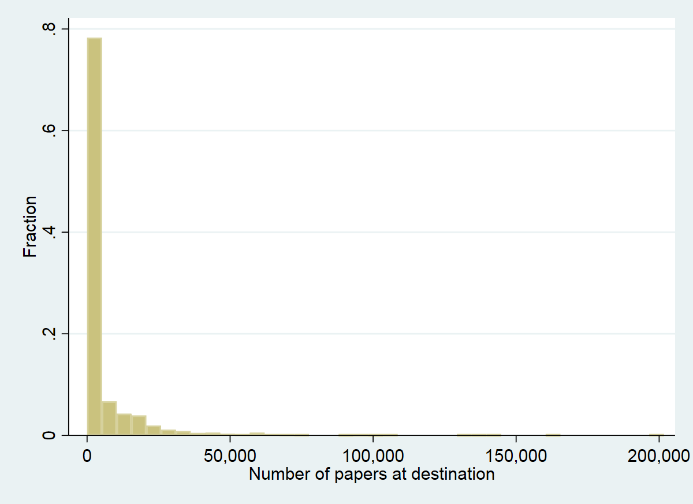

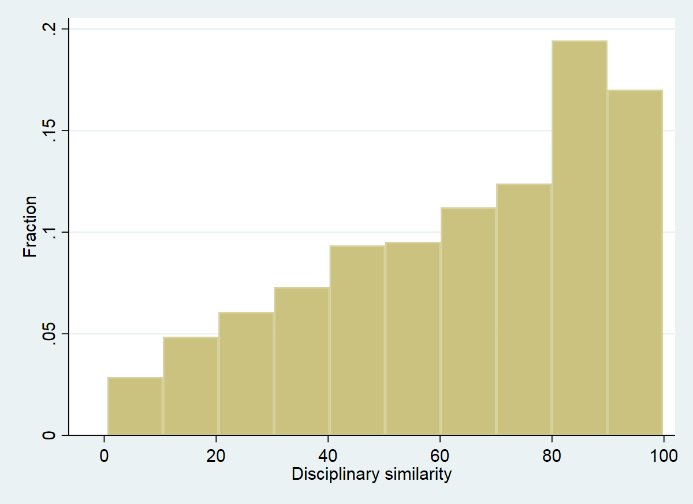


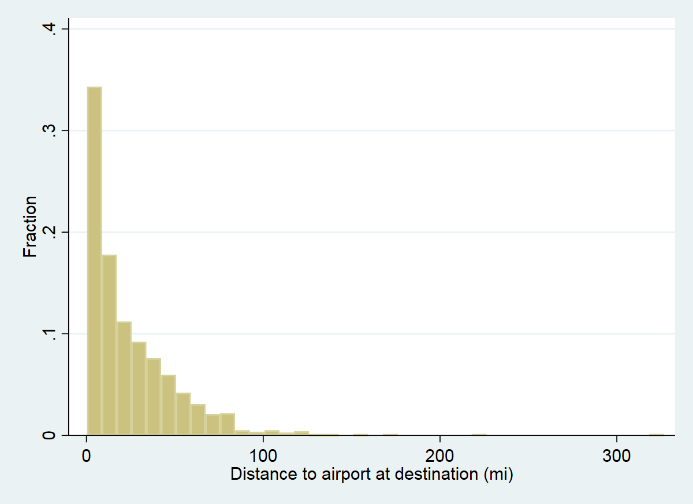

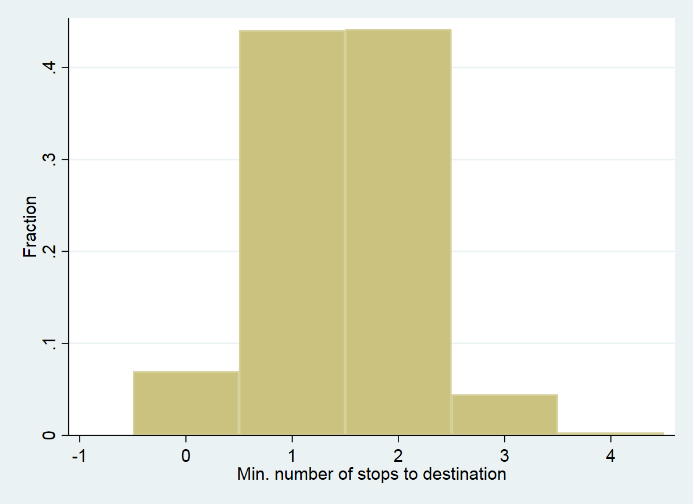


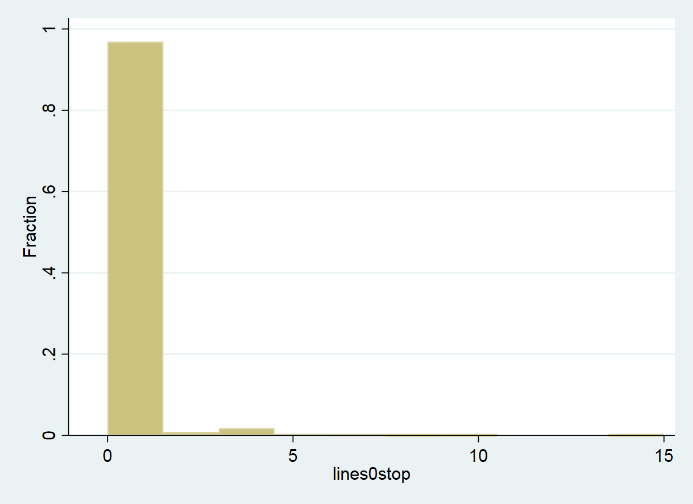

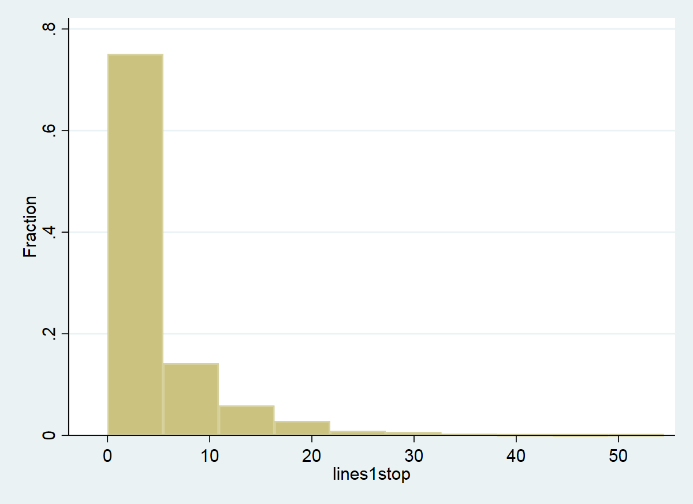


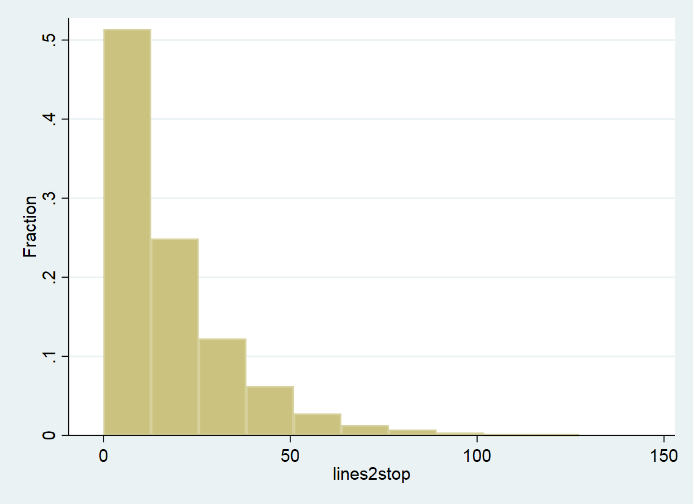

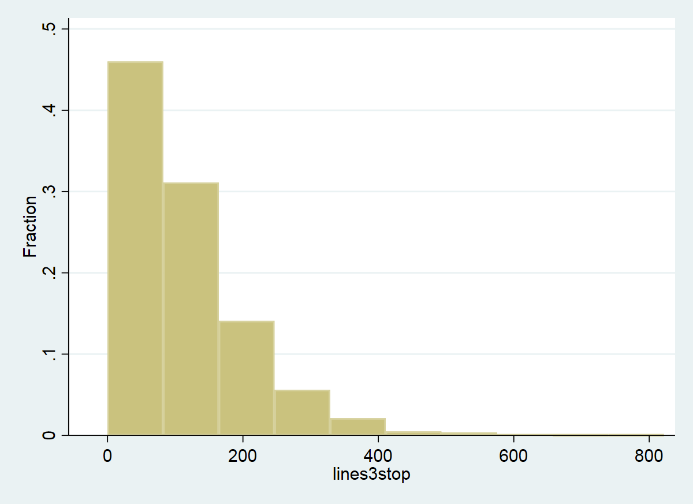


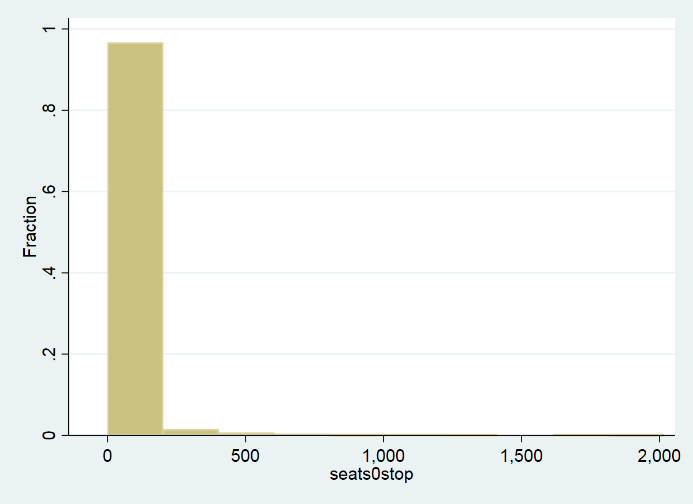

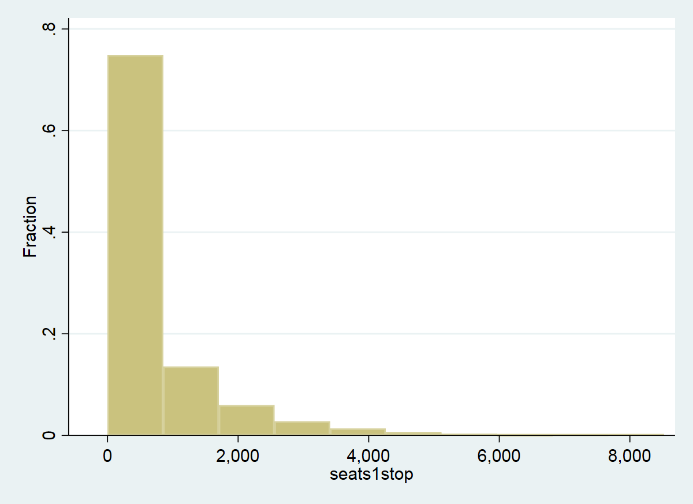


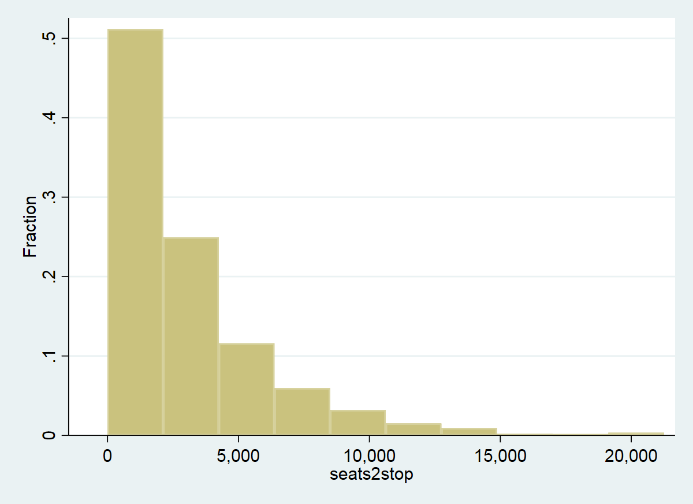

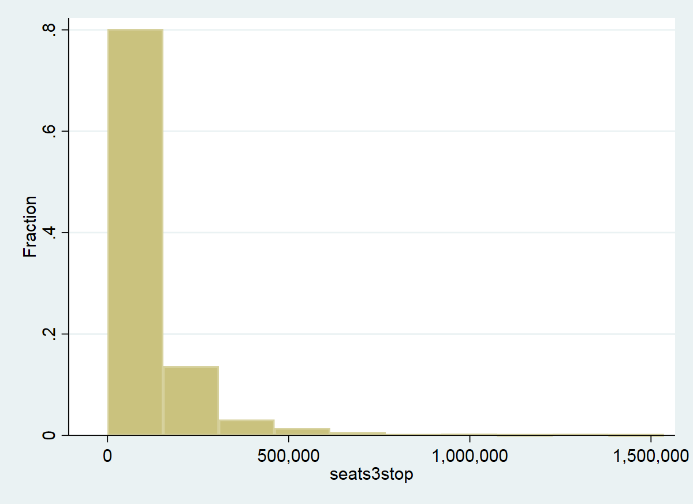


**Figure 7:** Histograms of variables used in the models presented in the article.

# Model specification and robustness checks

In this section, alternative model specifications are presented in order to justify specifications selected for the main analysis as well as to provide empirical evidence of robustness of the analysis. Specifically, we tested specifications that included additional variables in the 'inflate' part of the model. Models 1-9 (see the paper) were selected as a reference for this exercise. Results are presented in tables 1-3. Specifications in table 1 add ‘Disciplinary similarity’ variable in the count part. Specifications in table 2 add ‘Disciplinary similarity’ and ‘Geographical distance’ variables in the count part, while specifications in table 3 add ‘Disciplinary similarity’, ‘Geographical distance’, and air transport connectivity and accessibility variables. Generally speaking, adding more variables in the ‘inflate’ part does not cause significant changes in results. Coefficients of key variables, i.e., those measuring air transport connectivity and accessibility, are stable. In addition, the differences in matching models disclosed by AIC and BIC are small. As a consequence, the presented results provide the basis for preferring a simple model using in the 'inflate' part only one, theoretically justified, variable, i.e. the scientific potential of collaborators measured by the number of publications.

In the next step, various values of the exponent used to model geographical distance were tested. Exponent values from 1.1 to 3, in increments of 0.1, were applied to specification 9 (see table 4 from the article) – this model has the full specification in the 'inflate' part, i.e. it includes air transport connectivity and accessibility variables and has low AIC and BIC). The results of this exercise are shown in Table 4 below. In general, different variants of the ‘geographical distance’ exponent do not translate into significant differences in the degree of the fit of the model. The extreme values of AIC and BIC differ in a negligible way. In the case of AIC, the difference between the model with the highest value of this indicator and the model with the lowest result is only 3. In the case of BIC, it is 2.9. Coefficients of key variables, i.e., those measuring air transport connectivity and accessibility, are stable across compared models. Given the lack of substantial differences in the results generated by various specifications, it was decided to use exponent 2 (Geographical distance squared) in the main analyzes. This solution ensures consistency of the specifications presented in the article (squared terms used in other variables).

**Table 1.** Specification with additional variable in the ‘inflate’ part: ‘Disciplinary similarity’

| Dependent variable: Number of co-authored papers | (35) | (36) | (37) | (38) | (39) | (40) | (41) | (42) | (43) |
| --- | --- | --- | --- | --- | --- | --- | --- | --- | --- |
| **Count part** |  |  |  |  |  |  |  |  |  |
| Geographical distance  (thous mi) | -0.363*** | -0.315*** | -0.247*** | -0.271*** | -0.292*** | -0.292*** | -0.218*** | -0.246*** | -0.269*** |
| Geographical distance squared (thous mi) | 0.019*** | 0.015*** | 0.011*** | 0.013*** | 0.015*** | 0.012*** | 0.008** | 0.010*** | 0.012*** |
| Number of papers at destination | 0.116*** | 0.112*** | 0.107*** | 0.105*** | 0.105*** | 0.106*** | 0.099*** | 0.098*** | 0.098*** |
| Number of papers at destination squared | -0.000*** | -0.000*** | -0.000*** | -0.000*** | -0.000*** | -0.000*** | -0.000*** | -0.000*** | -0.000*** |
| Disciplinary similarity | 0.027*** | 0.026*** | 0.025*** | 0.023*** | 0.022*** | 0.024*** | 0.022*** | 0.020*** | 0.019*** |
| Disciplinary similarity squared | -0.000*** | -0.000*** | -0.000** | -0.000** | -0.000** | -0.000** | -0.000** | -0.000* | -0.000* |
| lines0stop |  | 0.299*** |  |  |  | 0.335*** |  |  |  |
| lines0stop squared |  | -0.023*** |  |  |  | -0.026*** |  |  |  |
| lines1stop |  |  | 0.073*** |  |  |  | 0.078*** |  |  |
| lines1stop squared |  |  | -0.001*** |  |  |  | -0.001*** |  |  |
| lines2stop |  |  |  | 0.028*** |  |  |  | 0.029*** |  |
| lines2stop squared |  |  |  | -0.000*** |  |  |  | -0.000*** |  |
| lines3stop |  |  |  |  | 0.005*** |  |  |  | 0.005*** |
| lines3stop squared |  |  |  |  | -0.000*** |  |  |  | -0.000*** |
| Distance to airport at destination (mi) |  |  |  |  |  | -0.011*** | -0.012*** | -0.012*** | -0.012*** |
| Constant | 0.950*** | 0.832*** | 0.496*** | 0.413** | 0.398** | 1.141*** | 0.795*** | 0.716*** | 0.700*** |
| **Inflate part** |  |  |  |  |  |  |  |  |  |
| Number of papers at destination | -4.224*** | -4.128*** | -4.082*** | -4.096*** | -4.090*** | -3.923*** | -3.855*** | -3.867*** | -3.865*** |
| Disciplinary similarity | 0.003 | 0.003 | 0.003 | 0.003 | 0.003 | 0.002 | 0.002 | 0.002 | 0.002 |
| Constant | -0.29 | -0.294 | -0.332* | -0.323* | -0.323* | -0.337* | -0.386* | -0.375* | -0.374* |
| Constant lnalpha | 0.804*** | 0.792*** | 0.773*** | 0.773*** | 0.771*** | 0.776*** | 0.753*** | 0.753*** | 0.752*** |
| **Statistics** |  |  |  |  |  |  |  |  |  |
| Observations | 8925 | 8925 | 8925 | 8925 | 8925 | 8925 | 8925 | 8925 | 8925 |
| AIC | 40808.6 | 40762.1 | 40629.4 | 40613.3 | 40601.1 | 40618.7 | 40460.4 | 40447.9 | 40434.6 |
| BIC | 40886.7 | 40854.4 | 40721.6 | 40705.6 | 40693.3 | 40718.1 | 40559.8 | 40547.3 | 40534 |

Significance levels: * p<0.05; ** p<0.01; *** p<0.001.

**Table 2.** Specification with additional variables in the ‘inflate’ part: ‘Disciplinary similarity’ and ‘Geographical distance’

| Dependent variable: Number of co-authored papers | (44) | (45) | (46) | (47) | (48) | (49) | (50) | (51) | (52) |
| --- | --- | --- | --- | --- | --- | --- | --- | --- | --- |
| **Count part** |  |  |  |  |  |  |  |  |  |
| Geographical distance  (thous mi) | -0.365*** | -0.317*** | -0.249*** | -0.273*** | -0.294*** | -0.295*** | -0.221*** | -0.250*** | -0.272*** |
| Geographical distance squared (thous mi) | 0.018*** | 0.015*** | 0.011*** | 0.013*** | 0.014*** | 0.012*** | 0.008** | 0.010*** | 0.012*** |
| Number of papers at destination | 0.117*** | 0.112*** | 0.107*** | 0.106*** | 0.106*** | 0.107*** | 0.100*** | 0.099*** | 0.099*** |
| Number of papers at destination squared | -0.000*** | -0.000*** | -0.000*** | -0.000*** | -0.000*** | -0.000*** | -0.000*** | -0.000*** | -0.000*** |
| Disciplinary similarity | 0.027*** | 0.026*** | 0.025*** | 0.023*** | 0.022*** | 0.024*** | 0.022*** | 0.020*** | 0.019*** |
| Disciplinary similarity squared | -0.000*** | -0.000*** | -0.000*** | -0.000** | -0.000** | -0.000** | -0.000** | -0.000* | -0.000* |
| lines0stop |  | 0.295*** |  |  |  | 0.330*** |  |  |  |
| lines0stop squared |  | -0.023*** |  |  |  | -0.026*** |  |  |  |
| lines1stop |  |  | 0.073*** |  |  |  | 0.079*** |  |  |
| lines1stop squared |  |  | -0.001*** |  |  |  | -0.001*** |  |  |
| lines2stop |  |  |  | 0.029*** |  |  |  | 0.030*** |  |
| lines2stop squared |  |  |  | -0.000*** |  |  |  | -0.000*** |  |
| lines3stop |  |  |  |  | 0.005*** |  |  |  | 0.005*** |
| lines3stop squared |  |  |  |  | -0.000*** |  |  |  | -0.000*** |
| Distance to airport at destination (mi) |  |  |  |  |  | -0.011*** | -0.012*** | -0.012*** | -0.012*** |
| Constant | 0.955*** | 0.838*** | 0.502*** | 0.417** | 0.402** | 1.150*** | 0.804*** | 0.722*** | 0.707*** |
| **Inflate part** |  |  |  |  |  |  |  |  |  |
| Number of papers at destination | -4.224*** | -4.128*** | -4.082*** | -4.096*** | -4.090*** | -3.923*** | -3.855*** | -3.867*** | -3.865*** |
| Disciplinary similarity | -4.585*** | -4.425*** | -4.412*** | -4.489*** | -4.493*** | -4.315*** | -4.290*** | -4.377*** | -4.392*** |
| Geographical distance  (thous mi) | 0.003 | 0.003 | 0.003 | 0.003 | 0.003 | 0.003 | 0.003 | 0.003 | 0.003 |
| Constant | -0.052* | -0.042 | -0.047* | -0.058* | -0.060* | -0.054* | -0.062* | -0.074** | -0.078** |
| Constant lnalpha | -0.126 | -0.161 | -0.182 | -0.142 | -0.136 | -0.165 | -0.192 | -0.146 | -0.135 |
| **Statistics** |  |  |  |  |  |  |  |  |  |
| Observations | 8925 | 8925 | 8925 | 8925 | 8925 | 8925 | 8925 | 8925 | 8925 |
| AIC | 40805.3 | 40760.6 | 40627 | 40609 | 40596.3 | 40615.5 | 40455.9 | 40440.9 | 40426.8 |
| BIC | 40890.5 | 40860 | 40726.3 | 40708.3 | 40695.6 | 40722 | 40562.3 | 40547.3 | 40533.2 |

Significance levels: * p<0.05; ** p<0.01; *** p<0.001.

**Table 3.** Specification with additional variables in the ‘inflate’ part: ‘Disciplinary similarity’, ‘Geographical distance’, and air transport connectivity and accessibility

| Dependent variable: Number of co-authored papers | (53) | (54) | (55) | (56) | (57) | (58) | (59) | (60) | (61) |
| --- | --- | --- | --- | --- | --- | --- | --- | --- | --- |
| **Count part** |  |  |  |  |  |  |  |  |  |
| Geographical distance  (thous mi) | -0.365*** | -0.319*** | -0.250*** | -0.275*** | -0.296*** | -0.295*** | -0.220*** | -0.250*** | -0.273*** |
| Geographical distance squared (thous mi) | 0.018*** | 0.015*** | 0.011*** | 0.013*** | 0.014*** | 0.012*** | 0.008** | 0.010*** | 0.012*** |
| Number of papers at destination | 0.117*** | 0.113*** | 0.107*** | 0.106*** | 0.106*** | 0.106*** | 0.100*** | 0.099*** | 0.099*** |
| Number of papers at destination squared | -0.000*** | -0.000*** | -0.000*** | -0.000*** | -0.000*** | -0.000*** | -0.000*** | -0.000*** | -0.000*** |
| Disciplinary similarity | 0.027*** | 0.026*** | 0.025*** | 0.023*** | 0.022*** | 0.024*** | 0.022*** | 0.020*** | 0.019*** |
| Disciplinary similarity squared | -0.000*** | -0.000*** | -0.000** | -0.000** | -0.000** | -0.000** | -0.000** | -0.000* | -0.000* |
| lines0stop |  | 0.279*** |  |  |  | 0.313*** |  |  |  |
| lines0stop squared |  | -0.022*** |  |  |  | -0.024*** |  |  |  |
| lines1stop |  |  | 0.072*** |  |  |  | 0.078*** |  |  |
| lines1stop squared |  |  | -0.001*** |  |  |  | -0.001*** |  |  |
| lines2stop |  |  |  | 0.028*** |  |  |  | 0.029*** |  |
| lines2stop squared |  |  |  | -0.000*** |  |  |  | -0.000*** |  |
| lines3stop |  |  |  |  | 0.005*** |  |  |  | 0.005*** |
| lines3stop squared |  |  |  |  | -0.000*** |  |  |  | -0.000*** |
| Distance to airport at destination (mi) |  |  |  |  |  | -0.012*** | -0.013*** | -0.013*** | -0.013*** |
| Constant | 0.955*** | 0.849*** | 0.514*** | 0.433*** | 0.423** | 1.177*** | 0.831*** | 0.754*** | 0.745*** |
| **Inflate part** |  |  |  |  |  |  |  |  |  |
| Number of papers at destination | -4.585*** | -4.436*** | -4.376*** | -4.453*** | -4.445*** | -4.609*** | -4.555*** | -4.633*** | -4.634*** |
| Disciplinary similarity | 0.003 | 0.003 | 0.003 | 0.003 | 0.003 | 0.003 | 0.003 | 0.003 | 0.003 |
| Geographical distance  (thous mi) | -0.052* | -0.053* | -0.056* | -0.066** | -0.069** | -0.070** | -0.073** | -0.086** | -0.092*** |
| lines0stop |  | -0.235 |  |  |  | -0.252 |  |  |  |
| lines1stop |  |  | -0.012 |  |  |  | -0.009 |  |  |
| lines2stop |  |  |  | -0.004 |  |  |  | -0.004 |  |
| lines3stop |  |  |  |  | -0.001 |  |  |  | -0.001 |
| Distance to airport at destination (mi) |  |  |  |  |  | -0.007** | -0.008** | -0.008** | -0.008** |
| Constant | -0.126 | -0.088 | -0.102 | -0.035 | 0.016 | 0.125 | 0.104 | 0.181 | 0.243 |
| Constant lnalpha | 0.809*** | 0.796*** | 0.778*** | 0.778*** | 0.777*** | 0.784*** | 0.760*** | 0.761*** | 0.760*** |
| **Statistics** |  |  |  |  |  |  |  |  |  |
| Observations | 8925 | 8925 | 8925 | 8925 | 8925 | 8925 | 8925 | 8925 | 8925 |
| AIC | 40805.3 | 40757.7 | 40628 | 40609.9 | 40596.6 | 40604.3 | 40448.1 | 40433.6 | 40418.8 |
| BIC | 40890.5 | 40864.1 | 40734.4 | 40716.4 | 40703.1 | 40724.9 | 40568.8 | 40554.3 | 40539.5 |

Significance levels: * p<0.05; ** p<0.01; *** p<0.001.

**Table 4.** Specification with different exponents for geographical distance – values from 1.1 to 2

| Dependent variable: Number of co-authored papers | (62) | (63) | (64) | (65) | (66) | (67) | (68) | (69) | (70) | (71) |
| --- | --- | --- | --- | --- | --- | --- | --- | --- | --- | --- |
| **Count part** |  |  |  |  |  |  |  |  |  |  |
| Geographical distance  (thous mi) | -1.038*** | -0.611*** | -0.469*** | -0.397*** | -0.355*** | -0.326*** | -0.306*** | -0.290*** | -0.278*** | -0.269*** |
| Geographical distance^1.1 | 0.698*** |  |  |  |  |  |  |  |  |  |
| Geographical distance^1.2 |  | 0.286*** |  |  |  |  |  |  |  |  |
| Geographical distance^1.3 |  |  | 0.157*** |  |  |  |  |  |  |  |
| Geographical distance^1.4 |  |  |  | 0.097*** |  |  |  |  |  |  |
| Geographical distance^1.5 |  |  |  |  | 0.064*** |  |  |  |  |  |
| Geographical distance^1.6 |  |  |  |  |  | 0.044*** |  |  |  |  |
| Geographical distance^1.7 |  |  |  |  |  |  | 0.031*** |  |  |  |
| Geographical distance^1.8 |  |  |  |  |  |  |  | 0.022*** |  |  |
| Geographical distance^1.9 |  |  |  |  |  |  |  |  | 0.016*** |  |
| Geographical distance^2 |  |  |  |  |  |  |  |  |  | 0.012*** |
| Number of papers at destination | 0.098*** | 0.098*** | 0.098*** | 0.098*** | 0.098*** | 0.098*** | 0.098*** | 0.098*** | 0.098*** | 0.098*** |
| Number of papers at destination squared | -0.000*** | -0.000*** | -0.000*** | -0.000*** | -0.000*** | -0.000*** | -0.000*** | -0.000*** | -0.000*** | -0.000*** |
| Disciplinary similarity | 0.019*** | 0.019*** | 0.019*** | 0.019*** | 0.019*** | 0.019*** | 0.019*** | 0.019*** | 0.019*** | 0.019*** |
| Disciplinary similarity squared | -0.000* | -0.000* | -0.000* | -0.000* | -0.000* | -0.000* | -0.000* | -0.000* | -0.000* | -0.000* |
| lines3stop | 0.005*** | 0.005*** | 0.005*** | 0.005*** | 0.005*** | 0.005*** | 0.005*** | 0.005*** | 0.005*** | 0.005*** |
| lines3stop squared | -0.000*** | -0.000*** | -0.000*** | -0.000*** | -0.000*** | -0.000*** | -0.000*** | -0.000*** | -0.000*** | -0.000*** |
| Distance to airport at destination (mi) | -0.012*** | -0.012*** | -0.012*** | -0.012*** | -0.012*** | -0.012*** | -0.012*** | -0.012*** | -0.012*** | -0.012*** |
| Constant | 0.796*** | 0.784*** | 0.774*** | 0.765*** | 0.758*** | 0.751*** | 0.746*** | 0.741*** | 0.736*** | 0.732*** |
| **Inflate part** |  |  |  |  |  |  |  |  |  |  |
| Number of papers at destination | -3.904*** | -3.905*** | -3.906*** | -3.907*** | -3.908*** | -3.909*** | -3.910*** | -3.911*** | -3.912*** | -3.912*** |
| Constant | -0.249** | -0.248** | -0.247** | -0.247** | -0.246** | -0.246** | -0.245** | -0.245** | -0.245** | -0.244** |
| Constant lnalpha | 0.752*** | 0.752*** | 0.752*** | 0.752*** | 0.752*** | 0.752*** | 0.752*** | 0.752*** | 0.752*** | 0.751*** |
| **Statistics** |  |  |  |  |  |  |  |  |  |  |
| Observations | 8925 | 8925 | 8925 | 8925 | 8925 | 8925 | 8925 | 8925 | 8925 | 8925 |
| AIC | 40435.1 | 40434.9 | 40434.7 | 40434.5 | 40434.3 | 40434.1 | 40433.9 | 40433.8 | 40433.6 | 40433.4 |
| BIC | 40527.3 | 40527.1 | 40526.9 | 40526.7 | 40526.6 | 40526.4 | 40526.2 | 40526 | 40525.9 | 40525.7 |

Significance levels: * p<0.05; ** p<0.01; *** p<0.001.

**Table 5.** Specification with different exponents for geographical distance – values from 2.1 to 3

| Dependent variable: Number of co-authored papers | (72) | (73) | (74) | (75) | (76) | (77) | (78) | (79) | (80) | (81) |
| --- | --- | --- | --- | --- | --- | --- | --- | --- | --- | --- |
| **Count part** |  |  |  |  |  |  |  |  |  |  |
| Geographical distance  (thous mi) | -0.261*** | -0.254*** | -0.249*** | -0.244*** | -0.240*** | -0.236*** | -0.233*** | -0.230*** | -0.227*** | -0.225*** |
| Geographical distance^2.1 | 0.009*** |  |  |  |  |  |  |  |  |  |
| Geographical distance^2.2 |  | 0.007*** |  |  |  |  |  |  |  |  |
| Geographical distance^2.3 |  |  | 0.005*** |  |  |  |  |  |  |  |
| Geographical distance^2.4 |  |  |  | 0.004*** |  |  |  |  |  |  |
| Geographical distance^2.5 |  |  |  |  | 0.003*** |  |  |  |  |  |
| Geographical distance^2.6 |  |  |  |  |  | 0.002*** |  |  |  |  |
| Geographical distance^2.7 |  |  |  |  |  |  | 0.002*** |  |  |  |
| Geographical distance^2.8 |  |  |  |  |  |  |  | 0.001*** |  |  |
| Geographical distance^2.9 |  |  |  |  |  |  |  |  | 0.001*** |  |
| Geographical distance^3 |  |  |  |  |  |  |  |  |  | 0.001*** |
| Number of papers at destination | 0.098*** | 0.098*** | 0.098*** | 0.098*** | 0.098*** | 0.098*** | 0.098*** | 0.098*** | 0.098*** | 0.098*** |
| Number of papers at destination squared | -0.000*** | -0.000*** | -0.000*** | -0.000*** | -0.000*** | -0.000*** | -0.000*** | -0.000*** | -0.000*** | -0.000*** |
| Disciplinary similarity | 0.019*** | 0.019*** | 0.019*** | 0.019*** | 0.018*** | 0.018*** | 0.018*** | 0.018*** | 0.018*** | 0.018*** |
| Disciplinary similarity squared | -0.000* | -0.000* | -0.000* | -0.000* | -0.000* | -0.000* | -0.000* | -0.000* | -0.000* | -0.000* |
| lines3stop | 0.005*** | 0.005*** | 0.005*** | 0.005*** | 0.005*** | 0.005*** | 0.005*** | 0.005*** | 0.005*** | 0.005*** |
| lines3stop squared | -0.000*** | -0.000*** | -0.000*** | -0.000*** | -0.000*** | -0.000*** | -0.000*** | -0.000*** | -0.000*** | -0.000*** |
| Distance to airport at destination (mi) | -0.012*** | -0.012*** | -0.012*** | -0.012*** | -0.012*** | -0.012*** | -0.012*** | -0.012*** | -0.012*** | -0.012*** |
| Constant | 0.729*** | 0.725*** | 0.722*** | 0.720*** | 0.717*** | 0.715*** | 0.712*** | 0.710*** | 0.708*** | 0.706*** |
| **Inflate part** |  |  |  |  |  |  |  |  |  |  |
| Number of papers at destination | -3.913*** | -3.914*** | -3.914*** | -3.915*** | -3.915*** | -3.915*** | -3.916*** | -3.916*** | -3.916*** | -3.916*** |
| Constant | -0.244** | -0.244** | -0.244** | -0.243** | -0.243** | -0.243** | -0.243** | -0.243** | -0.243** | -0.243** |
| Constant lnalpha | 0.751*** | 0.751*** | 0.751*** | 0.751*** | 0.751*** | 0.751*** | 0.751*** | 0.751*** | 0.751*** | 0.751*** |
| **Statistics** |  |  |  |  |  |  |  |  |  |  |
| Observations | 8925 | 8925 | 8925 | 8925 | 8925 | 8925 | 8925 | 8925 | 8925 | 8925 |
| AIC | 40433.3 | 40433.1 | 40433 | 40432.8 | 40432.7 | 40432.5 | 40432.4 | 40432.3 | 40432.2 | 40432.1 |
| BIC | 40525.5 | 40525.4 | 40525.2 | 40525.1 | 40524.9 | 40524.8 | 40524.7 | 40524.6 | 40524.5 | 40524.4 |

Significance levels: * p<0.05; ** p<0.01; *** p<0.001.

# Prediction experiment

A prediction experiment was performed to assess the fit of the models. For this purpose, the data set has been randomly divided into two parts—a training set of 80% of observations and a test set of 20% of observations. The training set was passed through all the model specifications discussed in the article (Tables 4-7, models 1-34). Then, based on the data from the test set, the predicted values ​​of the outcome variable, i.e. the number of co-authored papers, were calculated. In the next step, the predicted value of the outcome variable was compared to the actual value from the test set. This was done using a simple linear model (OLS) in which the left side of the equation is the actual number of co-authored papers, and the right side of the equation is the number of co-authored papers predicted (both variables are logarithmized to normalize their distributions).

Tables 6, 8, 10, and 12 present training sets estimates for models specified as in tables 4, 5, 6, and 7 in the main part of the article (The results based on the training set are numbered like the models in the main text of the article, but they are marked with a single quote sign). In general, the results are very similar to those calculated for the entire data set, which of course is in line with expectations, as the training set is a large subset of the entire data set. Tables 7, 9, 11, and 13 present OLS results of the actual number of co-authored articles regressed on the predicted number of co-authored articles in the test set (The results based on the test set are numbered like the models in the main text of the article, but they are marked with a double quote sign). The key statistic here is the coefficient of determination. The average R2 for all 34 models is 0.514 (The lowest R2 value is 0.459 and the highest is 0.57). These results testify to the relatively good fit of the model to the data: the predicted values ​​of the number of co-authored articles explain about half the variability of the actual number of co-authored articles.

**Table 6.** Training set estimates – for models specified as in Table 4 in the main part of the article

| Dependent variable: Number of co-authored papers | (1’) | (2’) | (3’) | (4’) | (5’) | (6’) | (7’) | (8’) | (9’) |
| --- | --- | --- | --- | --- | --- | --- | --- | --- | --- |
| **Count part** |  |  |  |  |  |  |  |  |  |
| Geographical distance  (thous mi) | -0.396*** | -0.356*** | -0.287*** | -0.308*** | -0.327*** | -0.327*** | -0.248*** | -0.275*** | -0.298*** |
| Geographical distance squared (thous mi) | 0.023*** | 0.020*** | 0.016*** | 0.017*** | 0.019*** | 0.017*** | 0.012*** | 0.014*** | 0.016*** |
| Number of papers at destination | 0.115*** | 0.111*** | 0.106*** | 0.104*** | 0.104*** | 0.104*** | 0.097*** | 0.096*** | 0.096*** |
| Number of papers at destination squared | -0.000*** | -0.000*** | -0.000*** | -0.000*** | -0.000*** | -0.000*** | -0.000*** | -0.000*** | -0.000*** |
| Disciplinary similarity | 0.028*** | 0.028*** | 0.027*** | 0.025*** | 0.024*** | 0.024*** | 0.022*** | 0.020*** | 0.020*** |
| Disciplinary similarity squared | -0.000** | -0.000** | -0.000** | -0.000** | -0.000** | -0.000* | -0.000* | 0 | 0 |
| lines0stop |  | 0.247*** |  |  |  | 0.303*** |  |  |  |
| lines0stop squared |  | -0.016 |  |  |  | -0.020** |  |  |  |
| lines1stop |  |  | 0.067*** |  |  |  | 0.077*** |  |  |
| lines1stop squared |  |  | -0.001*** |  |  |  | -0.001*** |  |  |
| lines2stop |  |  |  | 0.027*** |  |  |  | 0.029*** |  |
| lines2stop squared |  |  |  | -0.000*** |  |  |  | -0.000*** |  |
| lines3stop |  |  |  |  | 0.005*** |  |  |  | 0.005*** |
| lines3stop squared |  |  |  |  | -0.000*** |  |  |  | -0.000*** |
| Distance to airport at destination (mi) |  |  |  |  |  | -0.013*** | -0.015*** | -0.014*** | -0.014*** |
| Constant | 0.881*** | 0.787*** | 0.470*** | 0.374** | 0.351* | 1.156*** | 0.830*** | 0.733*** | 0.711*** |
| **Inflate part** |  |  |  |  |  |  |  |  |  |
| Number of papers at destination | -4.437*** | -4.345*** | -4.303*** | -4.321*** | -4.304*** | -4.128*** | -4.055*** | -4.071*** | -4.057*** |
| Constant | -0.047 | -0.043 | -0.062 | -0.076 | -0.081 | -0.149 | -0.181* | -0.196* | -0.202* |
| Constant lnalpha | 0.805*** | 0.795*** | 0.778*** | 0.778*** | 0.775*** | 0.774*** | 0.750*** | 0.750*** | 0.747*** |
| **Statistics** |  |  |  |  |  |  |  |  |  |
| Observations | 7113 | 7113 | 7113 | 7113 | 7113 | 7113 | 7113 | 7113 | 7113 |
| AIC | 32307.8 | 32279.3 | 32186.3 | 32168.9 | 32153.9 | 32129.3 | 32000.6 | 31986.2 | 31970.6 |
| BIC | 32376.5 | 32361.7 | 32268.8 | 32251.3 | 32236.3 | 32218.6 | 32090 | 32075.5 | 32059.9 |

Significance levels: * p<0.05; ** p<0.01; *** p<0.001.

**Table 7.** OLS results of the actual number of co-authored articles regressed on the predicted number of co-authored articles in the test set – for models specified as in Table 6 above

| Dependent variable: Number of co-authored papers (log) | (1’’) | (2’’) | (3’’) | (4’’) | (5’’) | (6’’) | (7’’) | (8’’) | (9’’) |
| --- | --- | --- | --- | --- | --- | --- | --- | --- | --- |
|  |  |  |  |  |  |  |  |  |  |
| Predicted number of co-authored papers (from model 1’) (log) | 0.709*** |  |  |  |  |  |  |  |  |
| Predicted number of co-authored papers (from model 2’) (log) |  | 0.718*** |  |  |  |  |  |  |  |
| Predicted number of co-authored papers (from model 3’) (log) |  |  | 0.700*** |  |  |  |  |  |  |
| Predicted number of co-authored papers (from model 4’) (log) |  |  |  | 0.694*** |  |  |  |  |  |
| Predicted number of co-authored papers (from model 5’) (log) |  |  |  |  | 0.689*** |  |  |  |  |
| Predicted number of co-authored papers (from model 6’) (log) |  |  |  |  |  | 0.693*** |  |  |  |
| Predicted number of co-authored papers (from model 7’) (log) |  |  |  |  |  |  | 0.669*** |  |  |
| Predicted number of co-authored papers (from model 8’) (log) |  |  |  |  |  |  |  | 0.664*** |  |
| Predicted number of co-authored papers (from model 9’) (log) |  |  |  |  |  |  |  |  | 0.659*** |
| Constant | 0.172*** | 0.163*** | 0.203*** | 0.216*** | 0.225*** | 0.228*** | 0.280*** | 0.291*** | 0.299*** |
| Observations | 1812 | 1812 | 1812 | 1812 | 1812 | 1812 | 1812 | 1812 | 1812 |
| R2 | 0.527 | 0.534 | 0.531 | 0.528 | 0.526 | 0.522 | 0.516 | 0.514 | 0.512 |

Significance levels: * p<0.05; ** p<0.01; *** p<0.001.

**Table 8.** Training set estimates – for models specified as in Table 5 in the main part of the article

| Dependent variable: Number of co-authored papers | (10’) | (11’) | (12’) | (13’) |
| --- | --- | --- | --- | --- |
| **Count part** |  |  |  |  |
| Geographical distance (thous mi) | -0.327*** | -0.292*** | -0.316*** | -0.329*** |
| Geographical distance squared (thous mi) | 0.017*** | 0.015*** | 0.016*** | 0.017*** |
| Number of papers at destination | 0.104*** | 0.097*** | 0.096*** | 0.098*** |
| Number of papers at destination squared | -0.000*** | -0.000*** | -0.000*** | -0.000*** |
| Disciplinary similarity | 0.023*** | 0.022*** | 0.019*** | 0.020*** |
| Disciplinary similarity squared | -0.000* | -0.000* | 0 | 0 |
| Seats0stop | 2.513*** |  |  |  |
| Seats0stop squared | -1.465*** |  |  |  |
| Seats1stop |  | 0.459*** |  |  |
| Seats1stop squared |  | -0.042*** |  |  |
| Seats2stop |  |  | 0.179*** |  |
| Seats2stop squared |  |  | -0.006*** |  |
| Seats3stop |  |  |  | 0.003*** |
| Seats3stop squared |  |  |  | -0.000*** |
| Distance to airport at destination (mi) | -0.013*** | -0.015*** | -0.014*** | -0.015*** |
| Constant | 1.159*** | 0.965*** | 0.867*** | 1.055*** |
| **Inflate part** |  |  |  |  |
| Number of papers at destination | -4.116*** | -4.058*** | -4.073*** | -4.116*** |
| Constant | -0.147 | -0.183* | -0.204* | -0.191* |
| Constant lnalpha | 0.773*** | 0.748*** | 0.745*** | 0.753*** |
| **Statistics** |  |  |  |  |
| Observations | 7113 | 7113 | 7113 | 7113 |
| AIC | 32124.5 | 31987.1 | 31957.3 | 31995.3 |
| BIC | 32213.8 | 32076.4 | 32046.6 | 32084.6 |

To ensure meaningful coefficients SeatsXstop variable is divided by 1000.

Significance levels: * p<0.05; ** p<0.01; *** p<0.001.

**Table 9.** OLS results of the actual number of co-authored articles regressed on the predicted number of co-authored articles in the test set – for models specified as in Table 8 above

| Dependent variable: Number of co-authored papers (log) | (10’’) | (11’’) | (12’’) | (13’’) |
| --- | --- | --- | --- | --- |
|  |  |  |  |  |
| Predicted number of co-authored papers (from model 10’) (log) | 0.694*** |  |  |  |
| Predicted number of co-authored papers (from model 11’) (log) |  | 0.663*** |  |  |
| Predicted number of co-authored papers (from model 12’) (log) |  |  | 0.658*** |  |
| Predicted number of co-authored papers (from model 13’) (log) |  |  |  | 0.652*** |
| Constant | 0.228*** | 0.290*** | 0.304*** | 0.303*** |
| Observations | 1812 | 1812 | 1812 | 1812 |
| R2 | 0.521 | 0.513 | 0.511 | 0.508 |

Significance levels: * p<0.05; ** p<0.01; *** p<0.001.

**Table 10.** Training set estimates – for models specified as in Table 6 in the main part of the article

| Dependent variable:  Number of co-authored papers | **Full dataset** | **ASU** | **IUB** | **IUPUI** | **UMICH** |
| --- | --- | --- | --- | --- | --- |
|  | (14’) | (15’) | (16’) | (17’) | (18’) |
| **Count part** |  |  |  |  |  |
| Geographical distance (thous mi) | -0.176*** | -0.416*** | -0.261*** | -0.501*** | -0.168** |
| Geographical distance squared (thous mi) | 0.007* | 0.022** | 0.016* | 0.043*** | 0.004 |
| Number of papers at destination | 0.100*** | 0.103*** | 0.107*** | 0.084*** | 0.131*** |
| Number of papers at destination squared | -0.000*** | -0.000*** | -0.001*** | -0.000*** | -0.001*** |
| Disciplinary similarity | 0.019*** | 0.042** | -0.052** | 0.015* | 0.042*** |
| Disciplinary similarity squared | 0 | -0.000* | 0.000** | 0 | -0.000*** |
| Minimum number of stops to reach destination (compared to direct flight): |  |  |  |  |  |
| 1 stop | -0.662*** | -0.313* | -0.214 | -0.855*** | -0.330** |
| 2 stops | -1.252*** | -0.408* | -0.776* | -1.122*** | -0.513*** |
| 3 stops | -1.509*** | -1.305*** | -1.240** | -0.798* | -0.301 |
| Distance to airport at destination (mi) | -0.014*** | -0.011*** | -0.011*** | -0.016*** | -0.014*** |
| Constant | 1.837*** | 1.208* | 3.245*** | 2.211*** | 1.274*** |
| **Inflate part** |  |  |  |  |  |
| Number of papers at destination | -4.174*** | -3.963*** | -5.214*** | -2.071*** | 0.019 |
| Constant | -0.173* | 0.106 | 0.735*** | 0.452** | -16.861 |
| Constant lnalpha | 0.741*** | 0.582*** | 0.634*** | 0.481*** | 0.643*** |
| **Statistics** |  |  |  |  |  |
| Observations | 7097 | 1803 | 1735 | 1794 | 1765 |
| AIC | 31925.3 | 7599.7 | 6433 | 6389.4 | 10735.5 |
| BIC | 32021.4 | 7676.7 | 6509.4 | 6466.3 | 10812.1 |

Significance levels: * p<0.05; ** p<0.01; *** p<0.001.

**Table 11.** OLS results of the actual number of co-authored articles regressed on the predicted number of co-authored articles in the test set – for models specified as in Table 10 above

| Dependent variable: Number of co-authored papers (log) | (14’’) | (15’’) | (16’’) | (17’’) | (18’’) |
| --- | --- | --- | --- | --- | --- |
|  |  |  |  |  |  |
| Predicted number of co-authored papers (from model 14’) (log) | 0.670*** |  |  |  |  |
| Predicted number of co-authored papers (from model 15’) (log) |  | 0.622*** |  |  |  |
| Predicted number of co-authored papers (from model 16’) (log) |  |  | 0.658*** |  |  |
| Predicted number of co-authored papers (from model 17’) (log) |  |  |  | 0.587*** |  |
| Predicted number of co-authored papers (from model 18’) (log) |  |  |  |  | 0.769*** |
| Observations | 1810 | 438 | 487 | 429 | 456 |
| R2 | 0.52 | 0.502 | 0.556 | 0.462 | 0.526 |

Significance levels: * p<0.05; ** p<0.01; *** p<0.001.

**Table 12.** Training set estimates – for models specified as in Table 7 in the main part of the article

| Dependent variable: Number of co-authored papers | **ASU** | | | | **IUB** | | | | **IUPUI** | | | | **UMICH** | | | |
| --- | --- | --- | --- | --- | --- | --- | --- | --- | --- | --- | --- | --- | --- | --- | --- | --- |
|  | (19’) | (20’) | (21’) | (22’) | (23’) | (24’) | (25’) | (26’) | (27’) | (28’) | (29’) | (30’) | (31’) | (32’) | (33’) | (34’) |
| **Count part** |  |  |  |  |  |  |  |  |  |  |  |  |  |  |  |  |
| Geographical distance (thous mi) | -0.451*** | -0.442*** | -0.432*** | -0.465*** | -0.398*** | -0.336*** | -0.378*** | -0.388*** | -0.568*** | -0.504*** | -0.545*** | -0.583*** | -0.247*** | -0.239*** | -0.243*** | -0.254*** |
| Geographical distance squared (thous mi) | 0.025*** | 0.024*** | 0.022*** | 0.026*** | 0.026*** | 0.021*** | 0.024*** | 0.025*** | 0.047*** | 0.044*** | 0.046*** | 0.049*** | 0.011* | 0.01 | 0.01 | 0.011* |
| Number of papers at destination | 0.103*** | 0.101*** | 0.099*** | 0.103*** | 0.109*** | 0.104*** | 0.101*** | 0.104*** | 0.084*** | 0.080*** | 0.079*** | 0.084*** | 0.128*** | 0.123*** | 0.122*** | 0.124*** |
| Number of papers at destination squared | -0.000*** | -0.000*** | -0.000*** | -0.000*** | -0.001*** | -0.001*** | -0.001*** | -0.001*** | -0.000*** | -0.000*** | -0.000*** | -0.000*** | -0.001*** | -0.001*** | -0.001*** | -0.001*** |
| Disciplinary similarity | 0.044** | 0.043** | 0.039** | 0.044** | -0.072*** | -0.064*** | -0.062** | -0.065*** | 0.016* | 0.014 | 0.013 | 0.013 | 0.040*** | 0.039*** | 0.038*** | 0.039*** |
| Disciplinary similarity squared | -0.000** | -0.000** | -0.000* | -0.000** | 0.001*** | 0.001*** | 0.001*** | 0.001*** | 0 | 0 | 0 | 0 | -0.000*** | -0.000*** | -0.000*** | -0.000*** |
| Seats0stop | 1.141* |  |  |  | 1.885 |  |  |  | 4.264** |  |  |  | 1.217 |  |  |  |
| Seats0stop squared | -0.489 |  |  |  | -1.912 |  |  |  | -3.492 |  |  |  | -0.956 |  |  |  |
| Seats1stop |  | 0.160* |  |  |  | 0.569*** |  |  |  | 0.396** |  |  |  | 0.243*** |  |  |
| Seats1stop squared |  | -0.01 |  |  |  | -0.123** |  |  |  | -0.037 |  |  |  | -0.022* |  |  |
| Seats2stop |  |  | 0.106*** |  |  |  | 0.199*** |  |  |  | 0.118* |  |  |  | 0.108*** |  |
| Seats2stop squared |  |  | -0.004* |  |  |  | -0.012* |  |  |  | -0.003 |  |  |  | -0.004* |  |
| Seats3stop |  |  |  | 0.001 |  |  |  | 0.004*** |  |  |  | 0.002* |  |  |  | 0.001*** |
| Seats3stop squared |  |  |  | 0 |  |  |  | -0.000*** |  |  |  | 0 |  |  |  | 0 |
| Distance to airport at destination (mi) | -0.011*** | -0.012*** | -0.012*** | -0.011*** | -0.010*** | -0.011*** | -0.011*** | -0.012*** | -0.016*** | -0.017*** | -0.017*** | -0.017*** | -0.014*** | -0.015*** | -0.014*** | -0.014*** |
| Constant | 0.863 | 0.839 | 0.791 | 0.864 | 3.663*** | 3.155*** | 3.006*** | 3.296*** | 1.367*** | 1.121*** | 1.123*** | 1.345*** | 1.152*** | 1.062*** | 0.963*** | 1.090*** |
| **Inflate part** |  |  |  |  |  |  |  |  |  |  |  |  |  |  |  |  |
| Number of papers at destination | -3.899*** | -3.872*** | -3.891*** | -3.889*** | -5.230*** | -5.233*** | -5.215*** | -5.224*** | -1.985*** | -2.058*** | -2.036*** | -2.131*** | -5.453 | -5.231 | -5.163 | -5.192 |
| Constant | 0.121 | 0.105 | 0.092 | 0.108 | 0.795*** | 0.761*** | 0.746*** | 0.755*** | 0.461*** | 0.427** | 0.420** | 0.439** | -1.819*** | -1.833*** | -1.851*** | -1.838*** |
| Constant lnalpha | 0.580*** | 0.580*** | 0.575*** | 0.585*** | 0.648*** | 0.640*** | 0.637*** | 0.642*** | 0.474*** | 0.483*** | 0.483*** | 0.504*** | 0.570*** | 0.558*** | 0.553*** | 0.561*** |
| **Statistics** |  |  |  |  |  |  |  |  |  |  |  |  |  |  |  |  |
| Observations | 1806 | 1806 | 1806 | 1806 | 1741 | 1741 | 1741 | 1741 | 1799 | 1799 | 1799 | 1799 | 1767 | 1767 | 1767 | 1767 |
| AIC | 7608.3 | 7605.3 | 7595.9 | 7609.5 | 6471.5 | 6453.2 | 6449.1 | 6453 | 6392.6 | 6386.5 | 6387.9 | 6400.7 | 10737.9 | 10717.1 | 10709 | 10722.1 |
| BIC | 7679.8 | 7676.8 | 7667.4 | 7681 | 6542.5 | 6524.2 | 6520.1 | 6524 | 6464 | 6457.9 | 6459.3 | 6472.1 | 10809.1 | 10788.3 | 10780.2 | 10793.3 |

To ensure meaningful coefficients SeatsXstop variable is divided by 1000.

Significance levels: * p<0.05; ** p<0.01; *** p<0.001.

**Table 13.** OLS results of the actual number of co-authored articles regressed on the predicted number of co-authored articles in the test set – for models specified as in Table 12 above

| Dependent variable: Number of co-authored papers (log) | (19’’) | (20’’) | (21’’) | (22’’) | (23’’) | (24’’) | (25’’) | (26’’) | (27’’) | (28’’) | (29’’) | (30’’) | (31’’) | (32’’) | (33’’) | (34’’) |
| --- | --- | --- | --- | --- | --- | --- | --- | --- | --- | --- | --- | --- | --- | --- | --- | --- |
|  |  |  |  |  |  |  |  |  |  |  |  |  |  |  |  |  |
| Predicted number of co-authored papers (from model 19’) (log) | 0.625*** |  |  |  |  |  |  |  |  |  |  |  |  |  |  |  |
| Predicted number of co-authored papers (from model 20’) (log) |  | 0.617*** |  |  |  |  |  |  |  |  |  |  |  |  |  |  |
| Predicted number of co-authored papers (from model 21’) (log) |  |  | 0.606*** |  |  |  |  |  |  |  |  |  |  |  |  |  |
| Predicted number of co-authored papers (from model 22’) (log) |  |  |  | 0.618*** |  |  |  |  |  |  |  |  |  |  |  |  |
| Predicted number of co-authored papers (from model 23’) (log) |  |  |  |  | 0.703*** |  |  |  |  |  |  |  |  |  |  |  |
| Predicted number of co-authored papers (from model 24’) (log) |  |  |  |  |  | 0.679*** |  |  |  |  |  |  |  |  |  |  |
| Predicted number of co-authored papers (from model 25’) (log) |  |  |  |  |  |  | 0.677*** |  |  |  |  |  |  |  |  |  |
| Predicted number of co-authored papers (from model 26’) (log) |  |  |  |  |  |  |  | 0.661*** |  |  |  |  |  |  |  |  |
| Predicted number of co-authored papers (from model 27’) (log) |  |  |  |  |  |  |  |  | 0.585*** |  |  |  |  |  |  |  |
| Predicted number of co-authored papers (from model 28’) (log) |  |  |  |  |  |  |  |  |  | 0.569*** |  |  |  |  |  |  |
| Predicted number of co-authored papers (from model 29’) (log) |  |  |  |  |  |  |  |  |  |  | 0.565*** |  |  |  |  |  |
| Predicted number of co-authored papers (from model 30’) (log) |  |  |  |  |  |  |  |  |  |  |  | 0.565*** |  |  |  |  |
| Predicted number of co-authored papers (from model 31’) (log) |  |  |  |  |  |  |  |  |  |  |  |  | 0.780*** |  |  |  |
| Predicted number of co-authored papers (from model 32’) (log) |  |  |  |  |  |  |  |  |  |  |  |  |  | 0.761*** |  |  |
| Predicted number of co-authored papers (from model 33’) (log) |  |  |  |  |  |  |  |  |  |  |  |  |  |  | 0.757*** |  |
| Predicted number of co-authored papers (from model 34’) (log) |  |  |  |  |  |  |  |  |  |  |  |  |  |  |  | 0.760*** |
| Constant | 0.410*** | 0.422*** | 0.437*** | 0.420*** | 0.501*** | 0.535*** | 0.541*** | 0.547*** | 0.516*** | 0.543*** | 0.546*** | 0.532*** | -0.061 | -0.016 | -0.004 | -0.017 |
| Observations | 438 | 438 | 438 | 438 | 487 | 487 | 487 | 487 | 430 | 430 | 430 | 430 | 457 | 457 | 457 | 457 |
| R2 | 0.506 | 0.499 | 0.491 | 0.499 | 0.57 | 0.563 | 0.564 | 0.549 | 0.459 | 0.449 | 0.448 | 0.448 | 0.531 | 0.525 | 0.525 | 0.521 |

Significance levels: * p<0.05; ** p<0.01; *** p<0.001.

# Expertise Profiles: Science Maps

Global maps of science are generated through scientific analysis of large-scale scholarly data sets (e.g., publication, funding, and patent data) in an effort to connect and make sense of the bits and pieces of knowledge they contain, see the *Atlas of Science* (Börner, 2010) and the Places & Spaces: Mapping Science exhibit at <http://scimaps.org>. The UCSD Map of Science is the most comprehensive map in existence today (Börner et al, 2012). It organizes and visually represents 554 subdisciplines of science and their relationships to one another. Subdisciplines are grouped into 13 overarching disciplines that are color coded (red for medicine, green for biology, etc.) and labelled. Compared to the map of the world, disciplines resemble continents while subdisciplines can be seen as countries. Science maps can be used to gain overviews of “all-of-science” or of a specific subdiscipline. The default zoom level on each map will display the high-level aggregation of the data showing the 13 disciplines. Zooming in will break the disciplines into the 554 subdisciplines.

Using a journal name based or keyword based mapping process, data overlays can be computed. For example, expertise profiles for an institution are generated by reading a file with relevant publications, identifying unique journal names, and overlaying geometric symbols such as circles atop the subdiscipline(s) that are associated with each journal. Multiple journals might be associated with a subdiscipline requiring aggregation. Circle symbols are area size coded relative to the number matching journals and colored according to the respective discipline.

Figure 8 shows all three maps of science: IUB (A), IUPUI (B), ASU (C), UMICH (D). For IUB, there are 8,665 papers published in 2,688 unique journals and 2,407 out of the 2,688 journals were mapped to 457 subdisciplines; for IUPUI there are 10,265 papers published in 2,610 journals; 2,391 out of these 2,610 journals were mapped to 438 subdisciplines; for ASU there are 13,647 papers published in 3,596 journals and 3,178 out of 3,596 journals were mapped to 493 subdisciplines; and UMich has 40,380 publications in 5,744 journals and 4,986 out of 5,744 records were mapped to 522 subdisciplines.

**A (IUB)**

**
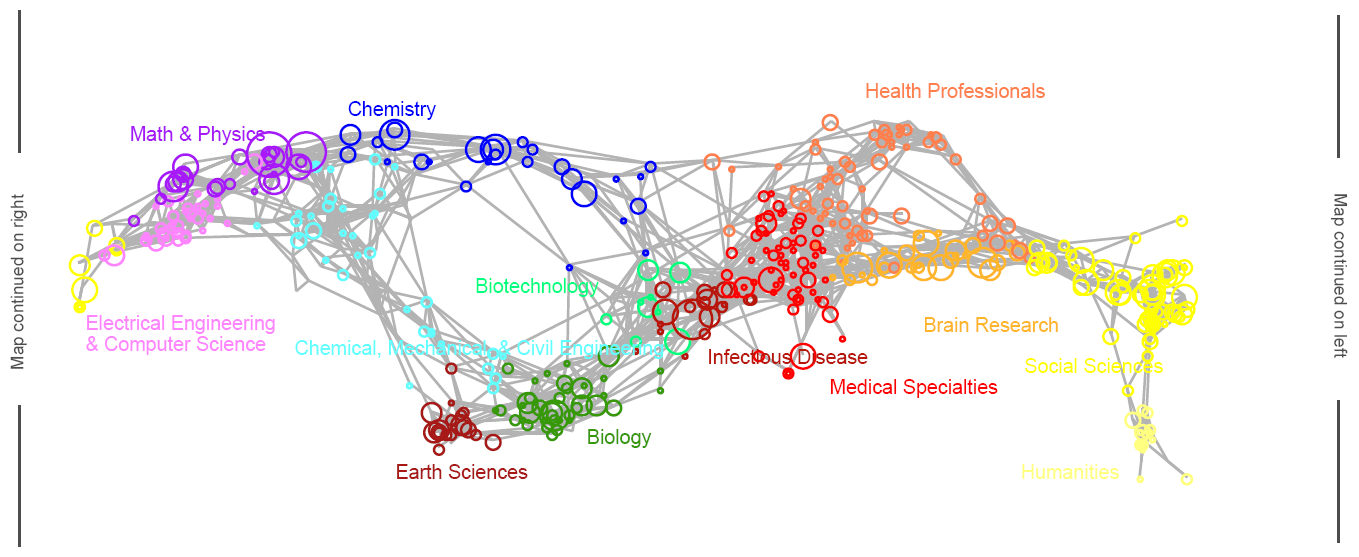
**

**B (IUPUI)**

**
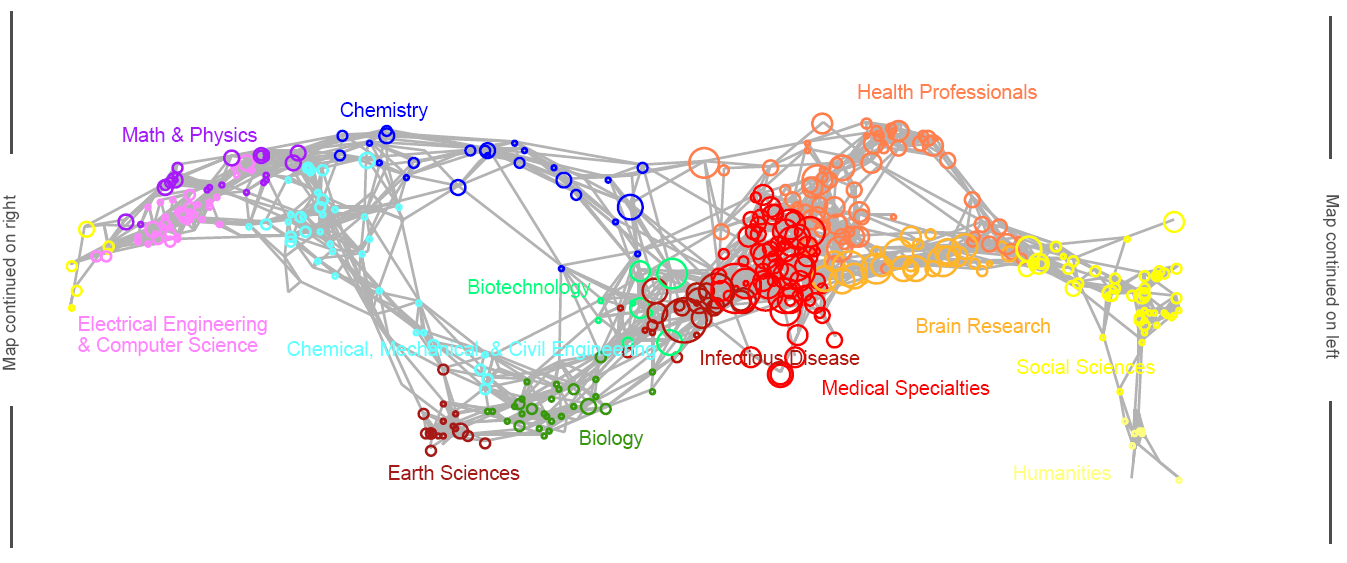
**

**C (ASU)**

**
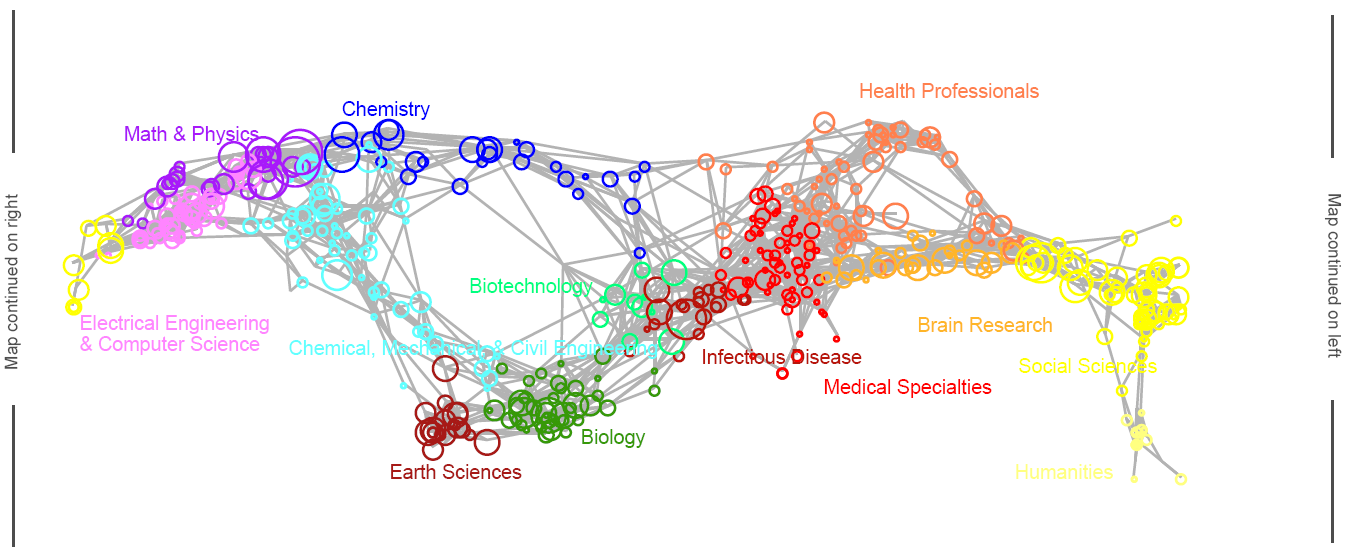
**

**D (UMICH)**

**
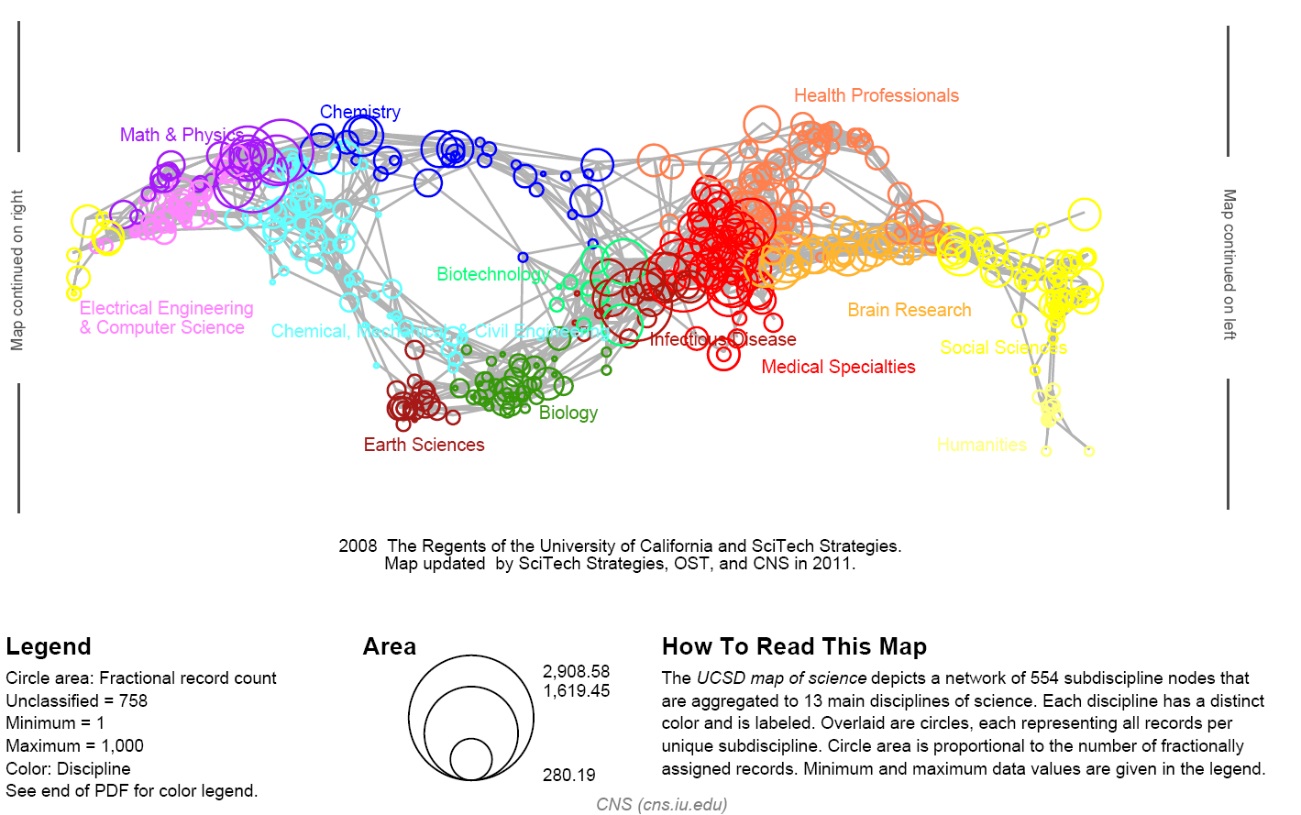
**

**Figure 8.** Science maps for all four universities showing differences in expertise profiles and productivity

As can be seen, the three universities have a rather different number of papers, published in smaller or larger numbers of unique journals. IUB has the smallest number of papers but comparably a rather large number of subdisciplines, see Table 14. As can be seen in Figure 8, IUPUI and UMich have a strong *Medial Specialties* focus—both have medical campus. All but IUPUI are strong in *Physics* and *Chemistry*.

**Table 14.** Number of papers, journals, and disciplines covered by the four universities

| **Disciplines** | **IUB** | **IUPUI** | **ASU** | **UMICH** | **IUB** | **IUPUI** | **ASU** | **UMICH** |
| --- | --- | --- | --- | --- | --- | --- | --- | --- |
| Biology | 661 | 76 | 995 | 1,218 | 7.6% | 0.7% | 7.3% | 3.2% |
| Biotechnology | 244 | 292 | 336 | 931 | 2.8% | 2.8% | 2.5% | 2.4% |
| Brain Research | 743 | 1,368 | 709 | 3,125 | 8.6% | 13.3% | 5.2% | 8.2% |
| Chemical, Mechanical, & Civil Engineering | 104 | 137 | 1,051 | 2,370 | 1.2% | 1.3% | 7.7% | 0.6% |
| Chemistry | 563 | 251 | 879 | 1,926 | 6.5% | 2.4% | 6.4% | 5.0% |
| Earth Sciences | 392 | 77 | 721 | 847 | 4.5% | 0.8% | 5.3% | 2.2% |
| Electrical Engineering & Computer Science | 279 | 153 | 975 | 1,522 | 3.2% | 1.5% | 7.1% | 4.0% |
| Health Professionals | 483 | 1,754 | 919 | 5,118 | 5.6% | 17.1% | 6.7% | 13.4% |
| Humanities | 105 | 11 | 50 | 147 | 1.2% | 0.1% | 0.4% | 0.4% |
| Infectious Diseases | 493 | 924 | 564 | 2,708 | 5.7% | 9.0% | 4.1% | 7.1% |
| Math & Physics | 1,322 | 206 | 1,712 | 4,353 | 15.3% | 2.0% | 12.5% | 11.4% |
| Medical Specialties | 517 | 3,681 | 468 | 8,314 | 6.0% | 35.9% | 3.4% | 21.7% |
| Social Sciences | 1,822 | 550 | 2,731 | 3,867 | 21.0% | 5.4% | 20.0% | 10.1% |
| Multiple Categories | 448 | 349 | 751 | 1,996 | 5.2% | 3.4% | 5.5% | 5.2% |
| Unclassified | 489 | 436 | 784 | 1,938 | 5.6% | 4.2% | 5.7% | 5.1% |
| **Total #papers** | **8,665** | **10,265** | **13,647** | **40,380** |  |  |  |  |
| **Total #journals** | **2,688** | **2,610** | **3,596** | **5,744** |  |  |  |  |
| **Total #subdisciplines** | **457** | **438** | **493** | **522** |  |  |  |  |

# Code instructions for result replication

The code for **data manipulation and data table generation** in written in JAVA. Source code and required input files are shared at <https://github.com/everyxs/FlightCoauthor>. The modeling and analysis is done in STATA and code and data is shared at the same GitHub repository under the folder [/STATA](https://github.com/everyxs/FlightCoauthor/tree/master/STATA).

The “FlightCoauthor.zip” contains a JAR package and a data folder set up for reproducing the data tables used in the main article. Java Runtime Environment (JRE) 7.0 or newer must be installed; all other required libraries are included in the JAR package. Once the zip content is extracted, run the JAR package by typing the following command under the extracted folder:

> Java –jar FlightCoaurhtor.jar

The data folder contains/produces the following files:

- Raw input files: “papers.csv”, “cityListJoin.csv”, “locations.csv”, “airports_metro.csv”, “airports_AP_joined_new.csv”, “routes.csv”, “planeWake.csv”, “legacyNodeTabel.csv”, “legacyEdgeTabel.csv”
- Intermediate data I/O file: “cityListMerged”, “massMatch0.csv”, “airportMergeMap.csv”, “collabEdges.csv”, “routesWeighted.csv”
- Final output data tables: “SelectCities[Nodes].csv”, “SelectCities[Edges].csv”

Since the Web of Science raw data is under Intellectual Property restrictions, we can only release a small sample in “papers.csv” for illustration purposes. As a result, the final edge table “SelectCities[Edges].csv” will have a very small “CollabPaper” column. For the actual “SelectCities[Edges].csv” table used in our analysis, please refer to “SelectCities[Edges]-full.csv”.

To recompile the sources files, Apache Commons lang3 and math3 Libraries are needed. The source code includes three classes, and their associated data files listed here:

- PrimaryCity.java, the main class where all other data is processed, including geo-coding, city merging, collaboration counting, etc.
  - Input files: “cityListJoin.csv”, “legacyNodeTabel.csv”, “locations.csv”, “papers.csv”, “collabEdges.csv”
  - Output files: “cityListMerged”, “massMatch0.csv”, “SelectCities[Edges].csv” (final edge data table)
- FlightNode.java, which handles airport merging and mapping with cities/metros.
  - Input files: “airports_metro.csv”, “airports_AP_joined_new.csv”, “massMatch0.csv”,
  - Output files: “airportMergeMap.csv”, “SelectCities[Nodes].csv” (final node data table)
- FlightEdge.java, which handles flight flow calculation and mapping with cities/metros.
  - Input files: “legacyEdgeTabel.csv”, “routes.csv”, “planeWake.csv”, “aiportMergeMap.csv”
  - Output files: “routesWeighted.csv”, “collabEdges.csv”

**Regression analyses** presented in the article can be replicated using STATA .do file: “AirSciColl_replication.do”. STATA version 14 or newer is needed to run the code. The STATA code uses analytical dataset named “AirSciColl_Analitical_Dataset.csv” that combines data tables described above. For convenience, the analitical dataset is limited to main variables used in the analysis. The code presented in “AirSciColl_replication.do” also allows reproduction of tables 1-7 and figure 3 from the main body of the article, as well as tables 1-13 from this supporting information document.

1. <http://iuni.iu.edu/resources/web-of-science> [↑](#footnote-ref-1)
2. <http://wiki.cns.iu.edu/pages/viewpage.action?pageId=1245861> [↑](#footnote-ref-2)
3. 2 <http://openflights.org/data.html>. [↑](#footnote-ref-3)
4. 9 <http://www.flugzeuginfo.net/table_accodes_iata_en.php> [↑](#footnote-ref-4)
