# Supplementary figures and images for: The impact of air transport availability on research collaboration: A case study of four universities
Source: PLoS One. 2020 Sep 4;15(9):e0238360. doi: 10.1371/journal.pone.0238360 (PMC7473522; doi:10.1371/journal.pone.0238360)

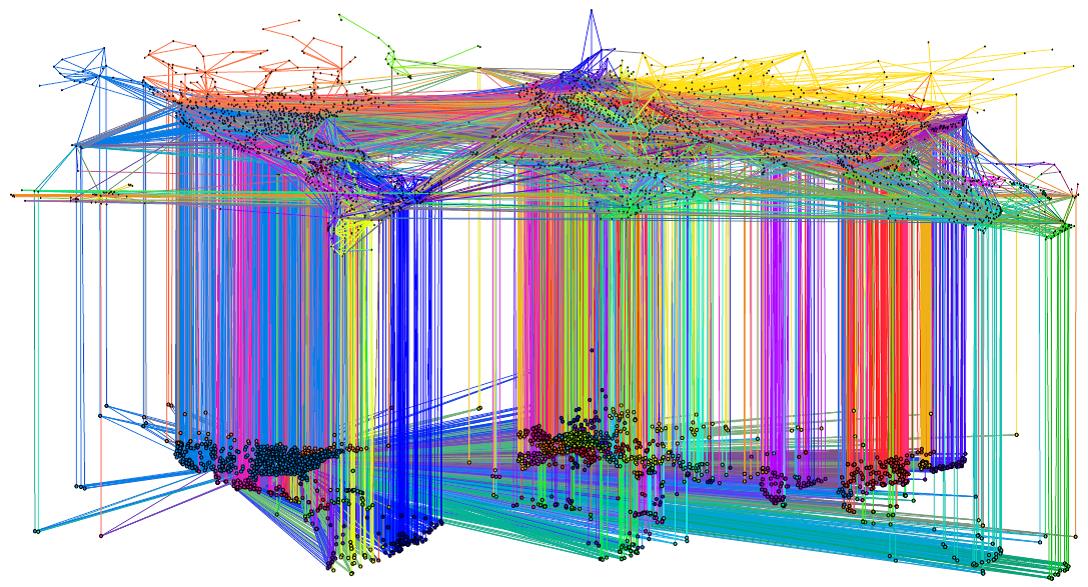

Supplement: S1 Fig — (JPG) [file pone.0238360.s002.jpg]
